# Analyzing Recent Coronary Heart Disease Mortality Trends in Tunisia between 1997 and 2009

**DOI:** 10.1371/journal.pone.0063202

**Published:** 2013-05-03

**Authors:** Olfa Saidi, Nadia Ben Mansour, Martin O’Flaherty, Simon Capewell, Julia A. Critchley, Habiba Ben Romdhane

**Affiliations:** 1 Cardiovascular Epidemiology and Prevention Research Laboratory, Faculty of Medicine of Tunis-Tunisia, Tunis, Tunisia; 2 Department of Public Health and Policy, University of Liverpool, Liverpool, United Kingdom; 3 Division of Population Health Sciences and Education, St George’s, University of London, London, United Kingdom; Inserm, France

## Abstract

**Background:**

In Tunisia, Cardiovascular Diseases are the leading causes of death (30%), 70% of those are coronary heart disease (CHD) deaths and population studies have demonstrated that major risk factor levels are increasing.

**Objective:**

To explain recent CHD trends in Tunisia between 1997 and 2009.

**Methods:**

Data Sources: Published and unpublished data were identified by extensive searches, complemented with specifically designed surveys.

**Analysis:**

Data were integrated and analyzed using the previously validated IMPACT CHD policy model. Data items included: (i)number of CHD patients in specific groups (including acute coronary syndromes, congestive heart failure and chronic angina)(ii) uptake of specific medical and surgical treatments, and(iii) population trends in major cardiovascular risk factors (smoking, total cholesterol, systolic blood pressure (SBP), body mass index (BMI), diabetes and physical inactivity).

**Results:**

CHD mortality rates increased by 11.8% for men and 23.8% for women, resulting in 680 additional CHD deaths in 2009 compared with the 1997 baseline, after adjusting for population change. Almost all (98%) of this rise was explained by risk factor increases, though men and women differed. A large rise in total cholesterol level in men (0.73 mmol/L) generated 440 additional deaths. In women, a fall (−0.43 mmol/L), apparently avoided about 95 deaths. For SBP a rise in men (4 mmHg) generated 270 additional deaths. In women, a 2 mmHg fall avoided 65 deaths. BMI and diabetes increased substantially resulting respectively in 105 and 75 additional deaths. Increased treatment uptake prevented about 450 deaths in 2009. The most important contributions came from secondary prevention following Acute Myocardial Infarction (AMI) (95 fewer deaths), initial AMI treatments (90), antihypertensive medications (80) and unstable angina (75).

**Conclusions:**

Recent trends in CHD mortality mainly reflected increases in major modifiable risk factors, notably SBP and cholesterol, BMI and diabetes. Current prevention strategies are mainly focused on treatments but should become more comprehensive.

## Introduction

The Eastern Mediterranean Region (EMR) has been recognized as a growing hot spot for Cardiovascular Diseases (CVD) and type2 diabetes. Projections of the growing burden exceed those of most other regions. About 47% of the region’s current burden of disease is due to non-communicable diseases (NCDs), and the Global Burden of Disease project have estimated that this proportion will rise to about 60% by the year 2020 [1]. Rates of coronary heart disease (CHD) will have increased by 160% in the region of Middle East and North Africa [2]. Meanwhile, mortality due to NCDs is decreasing in the developed world, namely, Western Europe, North America and Australia/New Zealand. This decline is driven largely by improved public health and medical access for a greater proportion of the population.

Tunisia is a Northern African country, located between Algeria and Libya, with a population of about ten million. It is typical amongst emerging South and East Mediterranean countries, having recently undergone a rapid economic development and is currently ranked 98^th^ out of 177 on the Human Development Index composite scale in 2009 [3].

Tunisia has experienced a crucial demographic transition, reflecting a sustained and integrated economical, social and health development. The global fertility rate is about 2 and the population is still young, with 24% under 15 years. However the population aged over 65 years is rapidly increasing, already exceeding 6% [4].Now, Tunisia is facing a rapidly growing burden of NCDs and CVDs are the leading causes of death accounting for almost 30%, 70% of those are CHD death [5], [6].

The epidemiological transition has been compounded by powerful environmental and behavioral changes. In particular, the adoption of new dietary habits and sedentary lifestyles, and the stress of urbanization and of working conditions may all lead to increases in major cardiovascular disease (CVDs) risk factors and mortality. Trends in conventional cardiovascular risk factors are well documented in Tunisia. Levels are dramatically high, especially in the coastal area [5], [7], [8]. In the meanwhile, health services, developed to tackle acute infectious diseases, appear to be unable to cope with the epidemiological situation and with the rises of the non-communicable diseases (NCDs).

This study aims to explain the substantial increase in CHD mortality between 1997 and 2009 in Tunisia, in order to inform future policy scenarios.

## Methods

The IMPACT model was previously validated in many developed countries with declining CHD trends and in China, where CHD trends increased [9].

The model is used to explore trends in the Middle East and specifically in Tunisia between 1997 and 2009.

Data on risk factor levels and current uptake levels of evidence based treatments were identified by extensive searches for published and unpublished data and complemented with specifically designed surveys. All data sources are detailed in Appendix S1 in File S1. The data required for the analysis was generally available for men and women aged over than 25 years in Tunisia for the period 1997–2009 with some exceptions amongst the oldest age groups (75 years and older). Data items used to populate the model included: (i) Patient numbers in specific CHD groups (including Myocardial Infarction Congestive Heart Failure, and Chronic Angina Pectoris, (ii) uptake of specific medical and surgical treatments, and (iii) population trends in major cardiovascular risk factors (smoking, total cholesterol, systolic blood pressure, body mass index, diabetes and physical inactivity).The main output of the model between 1997 and 2007 is the number of deaths prevented or postponed (DPPs) attributed to the change in treatment or risk factors level.

### Identification and Assessment of Relevant Data

Validated information on the Tunisian **population demographic changes** was obtained from Tunisian National Institute of Statistics for 1997 and 2009 [10], [4].

**The numbers of deaths for both years were** obtained from the National Public Health Institute - Tunisian Ministry of Public Health. CHD deaths are coded according to the 10th revision of International Classification of Diseases (ICD10), using the STIX software [4], [6], [10], [11].

**Population risk factors trend data** for 1997 and 2009 were obtained from national and regional epidemiological studies conducted in the community. The surveys were cross-sectional and the target population based on a nationally representative, stratified cluster sample of households according to the seven administrative regions of Tunisia and included both urban (65.9%) and rural areas (34.1% ) [7], [12]–[15].

**The numbers of hospital admissions with CHD in addition to treatment uptake** were obtained from the Tunisian Epidemiological Study “TEPS-ACS 2009” conducted in 2009 during the MedCHAMPS Project which included 5 of the most important Tunisian hospitals [16].

Data concerning secondary prevention was obtained from Premise I (Prevention of recurrence of Myocardial Infarction and Stroke) conducted by the Cardiovascular Epidemiology and Prevention Research Laboratory in 2002 and Premise II conducted in 2009 during the MedCHAMPS Project [17].

The number of patients undergoing Coronary Artery Bypass Grafting (CABG) and Angioplasty were obtained from the National Health Insurance Fund. The prevalence of Angina, Heart Attack survivors and Congestive Heart Failure in the community were estimated based on national health surveys and treatment uptake survey conducted by the Cardiovascular Epidemiology and Prevention Research Laboratory. We have access to the raw data for all these studies. Data on treatment uptake in the community was supported by systematically eliciting expert opinion.

### Efficacy of Therapeutic Interventions

We used recent meta-analyses and large randomised controlled trials. The Mant and Hicks approach was used to correct for polypharmacy. This approach consists to compare the relative sensitivity of measures of process and outcome to differences in quality of care or the hospital treatment of myocardial infarction basing on meta-analysis and large randomised controlled trials to estimate the impact that optimal use of these interventions would have on mortality in a typical district general hospital. [18].

### The Change in Coronary Heart Disease Deaths

First, the number of CHD deaths **expected** in 2009 was calculated by indirect age standardization assuming that 1997 mortality rates had persisted unchanged until 2009. The number of CHD deaths actually **observed** in 2009 was then subtracted. The difference between the two represents the rise or fall in coronary heart disease deaths (the number of deaths prevented or postponed) that the IMPACT model needed to explain.

### The Mortality Changes Attributed to Risk Factor Trends

The number of deaths prevented or postponed from changes in risk factors was estimated using two approaches. The regression β coefficients approach was used to quantify the population mortality impact of changes in those specific risk factors, measured as continuous variables, (systolic blood pressure, total cholesterol and body mass index - BMI). The second approach, population attributable risk fraction, was employed for categorical variables- diabetes, physical inactivity and smoking using Levin’s formula:
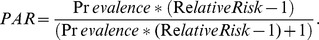



Details of the IMPACT model methodology have been published previously [19] and are detailed in appendix S2 in File S1.

### Estimating the Contribution of Medical and Surgical Treatments

The model aimed to include all medical and surgical treatments in 1997 (the base year) and 2009 (the final year). Treatment uptake data was not available for the year 1997 and was therefore estimated after consultation with cardiologists and experts working in both hospital and community at that time.

The mortality reduction for each treatment for the number of patients in each group, stratified by age and sex, was calculated as the relative mortality reduction reported in published meta-analyses multiplied by both; the age-specific case fatality observed in that group and the patient uptake (the proportion receiving that specific treatment (Appendix S1 in File S1). Survival benefit over a one-year time interval was used for all treatments.

### Treatment Adherence and Overlaps

Potential overlaps between different groups of patients were identified and appropriate adjustments were then made. Patient group calculations and assumptions are detailed in Appendix S2 in File S1.

Adherence (defined as the proportion of treated patients actually taking therapeutically effective levels of the prescribed medication) was assumed to be 100% among hospital patients, 70% among all symptomatic community patients, and 50% among asymptomatic community patients, based on the literature and expert opinion.

### Model Validation: Comparison with Observed Mortality Falls

The model **estimate** for the changes in deaths attributed to all treatments plus all risk factor changes was summed for men and women in each specific age group and then the model fit was compared with the **observed change** in mortality for that group.

### Sensitivity Analyses

Because of the uncertainties surrounding some of the values, multi-way sensitivity analyses using the Brigg’s analysis of extremes method was used [20].

## Results

Tunisia has approximately 10 million inhabitants: The population has grown from 9,211,000 in 1997 to10, 458,000 in 2009. We investigated the population aged over 25 years. For this age group, the number of men grew from 2,125,000 in 1997 to 2, 789,0000 in 2009 and from 2,142,000 to 2,910,000 for women.

In Tunisia, recent trends in coronary heart disease (CHD) mortality were complex.

The rise in age specific CHD mortality among women was twice as large as the observed among men aged over than 25 years between 1997 and 2009. It increased by 11.8% in men (from 70 to 87 per 100 000) and by 23.8% in women (from 28 to 41 per 100 000).The rise in mortality was observed primarily in men aged 55+ and women 65+, while younger men and women showed a decline of 5% (men 25–54) and 7% (women 45–64) (Table 1).

**Table 1:** Population sizes and death rates of CHD in Tunisia, 1997 and 2009.

Gendre	Age group	Population (1000)		Death (number)		Death rates per 100,000		% Change	
		1997	2009	1997	2009	1997	2009	(Crude)	(Weighted by pop)
**Men**	**25–34**	733	866	10	11	1	1	−11.1%	−3.5%
	**35–44**	549	670	34	82	6	12	100.3%	24.1%
	**45–54**	318	571	215	261	68	46	−32.2%	−6.6%
	**55–64**	261	330	334	426	128	129	0.9%	0.1%
	**65–74**	174	216	454	645	260	299	14.8%	1.1%
	**75–84**	61	95	333	713	543	753	38.7%	1.3%
	**> = 85**	29	41	112	290	387	702	81.4%	1.2%
	**Subtotal**	**2126**	**2789**	**1492**	**2429**	**70**	**87**	**24.0%**	**11.8%**
**Women**	**25–34**	751	908	0	3	0	0		
	**35–44**	546	723	12	29	2	4	85.0%	21.1%
	**45–54**	329	574	55	52	17	9	−46.0%	−9.1%
	**55–64**	264	348	118	91	45	26	−41.3%	−4.9%
	**65–74**	201	227	194	271	96	119	24.2%	1.9%
	**75–84**	57	92	175	464	309	502	62.8%	2.0%
	**> = 85**	28	39	60	294	215	757	252.9%	3.4%
	**Subtotal**	**2176**	**2911**	**614**	**1205**	**28**	**41**	**46.6%**	**23.8%**
**Total**		**4301**	**5700**	**2106**	**3633**	**49**	**64**	**30.2%**	

This resulted in 680 additional CHD deaths in 2009 compared with the number expected if 1997 mortality rates had persisted.

### Risk Factor Changes between 1997 and 2009 (Figure 1)

Changes in major cardiovascular risk factors together produced a best estimate of 665 more deaths (minimum estimate 354, maximum 905) (Table 2). The foremost contribution came from the rise in total cholesterol levels leading to an estimated 340 additional deaths (minimum 160, maximum 430). A large rise in men (0,73 mmol/L), generated approximately 440 additional deaths. However in women, a fall (−0,43 mmol/L), apparently avoided some 95 deaths. The increase in systolic blood pressure generated 270 additional deaths (minimum 125, maximum 295) in men reflecting a large rise (4 mmHg). In women, a fall (2 mmHg), resulted in approximately 65 fewer deaths. Increased mean BMI increased substantially in men (2 Kg/m^2^) causing 85 and in women (1 Kg/m^2^) causing 20 additional deaths. Diabetes prevalence increased from 12% to 17% between 1997 and 2009 resulting approximately 75 additional deaths (minimum approximately 50, maximum 110) respectively 65 and 10 in men and women. Favorable trends in physical inactivity and smoking prevented or postponed approximately 45 (minimum 25, maximum 65) deaths. Self-reported physical inactivity decreased from 98.4% to 85, 2% in between 1997 and 2009. This generated approximately 35 fewer deaths in men and 10 fewer in women. Smoking changes prevented approximately 15 (minimum 10, maximum 25) CHD deaths overall. A decrease in smoking resulted in some 55 fewer deaths in men, but 40 additional deaths in women were statistically attributed to a rise in smoking (Table 2).

**Table 2:** Deaths attributable to population risk factor changes in Tunisia 1997–2009.

RISK FACTORS	Risk factor level		Risk factor change		Deaths prevented or postponed (DPPs)			% of Total mortality change
	1997	2009	Absolute	Relative	Best	*Min*	*Max*	
**SMOKING (Total): %**	23.9%	22.7%	0.01	−0.05	**15**	*10*	*25*	**2.2%**
**Men**	47.2%	41.9%	0.05		55			
**Women**	1.5%	4.3%	−0.03		−40			
**SYSTOLIC BLOOD PRESSURE (Total): mm/HG**	130.41	131.03	−0.62	0.00	−**205**	−*125*	−*295*	−**30.8%**
**Men**	129.45	133.42	−3.97		−270			
**Women**	131.33	128.75	2.59		65			
**CHOLESTEROL (Total): mmol/l**	4.59	4.73	−0.14	−0.04	−**340**	−*160*	−*430*	−**51.1%**
**Men**	4.44	5.17	−0.73		−440			
**Women**	4.73	4.31	0.43		100			
**PHYSICAL INACTIVITY(Total): %**	98.44%	85.16%	0.13	−0.13	**45**	*30*	*65*	**6.7%**
**Men**	97.29%	81.62%	0.16		35			
**Women**	99.53%	88.54%	0.11		10			
**BMI (Total):Kg/m^2^**	26.03	27.47	−1.45	0.06	−**105**	*60*	*160*	−**15.8%**
**Men**	24.83	26.76	−1.94		−85			
**Women**	27.17	28.15	−0.98		−20			
**DIABETES (Total): %**	12.09%	17.26%	−0.05	0.43	−**75**	*50*	*110*	−**11.2%**
**Men**	11.99%	18.89%			−65			
**Women**	12.20%	15.70%			−10			
**Estimated total risk factor effects**					−**665**	−*135*	−*770*	−**97.8%**

**Figure 1:**
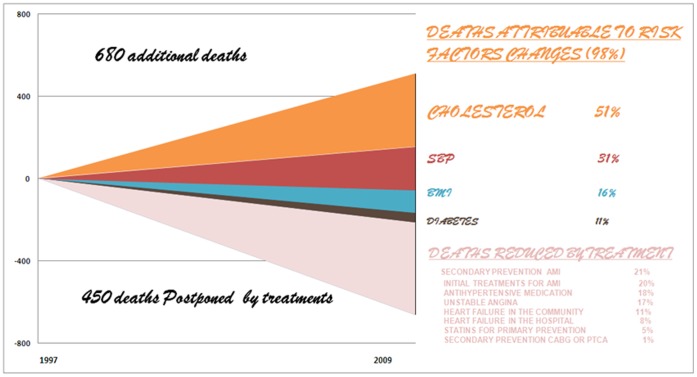
CHD Mortality Trends in Tunisia 1997–2009: additional deaths attributable to risk factor changes & deaths prevented or postponed by treatments.

### Medical and Surgical Treatments (Figure 1)

The model approximately estimated that 450 (minimum 180, maximum 1565) CHD deaths were prevented or postponed by medical and surgical treatments between 1997 and 2009. The biggest contributions came from secondary prevention following Acute Myocardial Infarction (95, (minimum 30, maximum 225), with ACE inhibitors, Beta blockers, statins, warfarin and aspirin being the main contributors in this group. Initial treatments for AMI explained approximately 90 (minimum 50, maximum 220) deaths prevented or postponed. Antihypertensive medications, treatments for unstable angina, as well as heart failure in the community and in hospital have contributed to a larger proportion of the mortality reductions; respectively estimated as 80 (minimum 10, maximum 450), 75 (minimum 35, maximum 175), 50 (minimum 15, maximum 115) and 35(minimum 10, maximum 85). Smaller contributions to DPPs were estimated from secondary prevention post angioplasty 5 deaths (minimum 1, maximum 10) (Table 3).

**Table 3:** Deaths prevented or postponed by medical & surgical treatments in TUNISIA in 2009.

	Treatmentuptake	Best DPPs	*Min DPPs*	*Max DPPs*
**INITIAL TREATMENTS FOR ACUTE MI**		**90**	***50***	***220***
Community CPR	5,0%	0	*0*	*1*
Hospital CPR	50,0%	12	*8*	*21*
Thrombolysis	20,4%	13	*5*	*25*
Aspirin	94,8%	46	*19*	*84*
Beta blockers	67,8%	8	*3*	*16*
ACE inhibitor	71,6%	16	*7*	*33*
PTCA (STEMI)	43,2%	41	*17*	*85*
CABG	4,0%	2	*1*	*5*
Cardiac rehabilitation	0,0%	0	*0*	*0*
**UNSTABLE ANGINA**		**75**	***35***	***175***
Aspirin&heparin	66,1%	59	*24*	*123*
Aspirin	29,0%	16	*7*	*32*
PG IIA/IIIB	1,1%	0	*0*	*1*
CABG surgery	4,2%	5	*2*	*11*
PTCA	5,0%	5	*2*	*10*
**SECONDARY PREVENTION FOLLOWING AMI**		**95**	***30***	***225***
Aspirin	91,9%	95	*31*	*224*
Beta blockers	45,7%	40	*13*	*90*
ACE inhibitors	54,0%	39	*13*	*98*
Statins	53,6%	39	*13*	*97*
Warfarin	0,0%	44	*14*	*109*
Rehabilitation including exercise	5,4%	0	*0*	*0*
**SECONDARY PREVENTION FOLLOWING CABG OR PTCA**		**5**	***1***	***10***
Aspirin	94,8%	2	*1*	*4*
Beta blockers	39,1%	1	*0*	*3*
ACE inhibitors	48,9%	1	*0*	*3*
Statins	72,0%	2	*1*	*5*
Warfarin	0,9%	0	*0*	*0*
Rehabilitation	0,0%	0	*0*	*0*
**HEART FAILURE IN THE HOSPITAL**		**35**	***10***	***90***
ACE inhibitors	20,0%	10	*3*	*30*
Beta blockers	50,0%	44	*12*	*131*
Spironolactone	25,0%	25	*8*	*62*
Aspirin	95,0%	48	*16*	*104*
Statins	0,0%	0	*0*	*0*
**HEART FAILURE IN THE COMMUNITY**		**50**	***15***	***115***
ACE inhibitor	10,0%	14	*4*	*42*
Beta blockers	21,7%	67	*22*	*167*
Spironolactone	7,1%	19	*6*	*48*
Aspirin	49,0%	71	*23*	*172*
Statins	21,8%	0	*0*	*0*
**STATINS FOR PRIMARY PREVENTION**		**20**	***5***	***210***
**ANTIHYPERTENSIVE MEDICATION**		**80**	***12***	***451***
TOTAL TREATMENT EFFECTS IN 2009		1170	*365*	*3515*
TOTAL TREATMENT EFFECTS IN 1997		720	*225*	*2150*
**INCREASE IN TREATMENT EFFECTS BETWEEN 1997 AND 2009**		**450**	***180***	***1565***

### Validation and Model Fit

In summary, when including risk factors and treatment data, the model explained approximately 45% of the total mortality increase in the Tunisian population between 1997 and 2009. The remaining percent was unexplained and might reflect data quality issues or changes in other, unmeasured risk factors. The model estimates of deaths were generally consistent with the observed deaths for almost all age groups. The exception was in the age groups 45**–**54 and 55**–**64 years where a higher number of deaths were expected for both men and women. Overall, the model fit was better for women than for men (Table 4).

**Table 4:** Model validation: estimated versus observed changes in CHD deaths in Tunisia between 1997 and 2009.

	OBSERVED CHANGE 1997–2009	MODEL ESTIMATES	Discrepancy	Model FIT
**M 25–34**	−1	1	−2	−100%
**M 35–44**	−49	−41	−7	84%
**M 45–54**	−47	124	−171	−265%
**M 55–64**	−92	−4	−88	4%
**M 65–74**	−191	−82	−107	43%
**M 75–84**	−380	−198	−181	52%
**M 85+**	−178	−130	−48	73%
**Total Men**	−**936**	−**330**	−**604**	**35%**
**F 25–34**	−3	−3	0	100%
**F 35–44**	−17	−14	−4	82%
**F 45–54**	3	45	−41	1500%
**F 55–64**	27	64	−38	237%
**F 65–74**	−77	−53	−25	68%
**F 75–84**	−289	−179	−110	62%
**F 85+**	−234	−211	−23	90%
**Total women**	−**591**	−**350**	−**241**	**60%**
**Total** **Men & Women**	−**1527**	−**680**	−**845**	**45%**

### Sensitivity Analyses (Figures 2 and 3)

**Figure 2:**
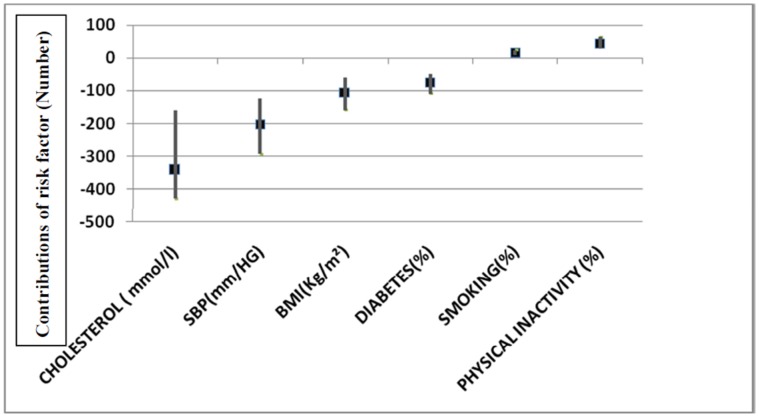
Proportional contributions of specific risk factor changes to trends in CHD deaths number in Tunisia 1997–2009- sensitivity analysis.

**Figure 3:**
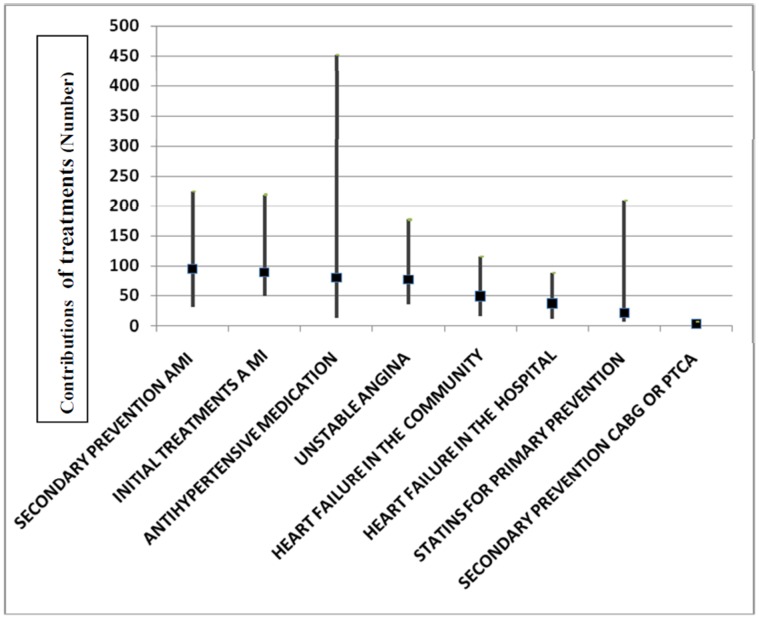
Proportional contributions of specific treatments to trends in CHD deaths number in Tunisia 1997–2009- sensitivity analysis.

Moreover, irrespective of whether best minimum or maximum estimates were used, the relative contributions remained relatively consistent (Figure 2 and 3). The largest part of the mortality increase was explained by large rises in total cholesterol levels, blood pressure, BMI and diabetes (Figure 2). Likewise, the principal mortality reductions from treatments consistently came from therapies for acute coronary syndromes, secondary prevention, heart failure and hypertension (Figure 3).

## Discussion

Coronary heart disease mortality increased by 17% in Tunisia between 1997 and 2009. Similar mortality rises have recently been observed in many other middle income countries [9], [21].

Almost all of this CHD mortality increase in Tunisia was explained by rises in major risk factors. With increased levels of development, come increased risk factors levels as well as improvements in uptake treatments. In fact, without improvements in treatments, the death rates would have been even higher.

### Cholesterol, Blood Pressure, Diet and Physical Activity

The biggest CHD mortality contribution came from the rises in total cholesterol and systolic blood pressure levels, but only among men. These differing gender trends can be partly explained by some dietary characteristics, such as fat and salt intakes which were significantly higher in men in the nutritional survey [13].

Rises in total cholesterol and systolic blood pressure levels generally reflect prominent lifestyle changes. As most developing countries, Tunisia has experienced a “hyper urbanization” that has improved health coverage, but has also introduced new lifestyles, such as sedentary habits and westernized diet [5] Nationally, the proportion of animal’s protein among the total protein intake has almost doubled during the last 3 decades (14% in 1975 to 27% in 2005) [22]. In fact, compared with rural areas Tunisian people in urban areas consume almost twice as much meat (42 Kg/person/year vs. 26 Kg/person/year) and milk (63 Kg/person/year vs. 38 Kg/person/year). Conversely, wheat consumption is almost 3 times higher in rural areas (129 vs.55 Kg/person/year).

A large meta-analysis estimated that a long term reduction in cholesterol concentration of 10%, which can be achieved by moderate dietary change, lowers the risk of ischemic heart disease by 30% at age of 60 years. The full benefits are achieved by 5 years [23]. Healthy diet therefore currently represents a missed opportunity for health promotion policies in Tunisia.

Increasing physical activity levels play a potentially important position in reducing cardiovascular risk factors [24]. However, a recent study conducted in an urban area of Tunis showed that more than the half of the population has low levels of physical activity [8].

Surprisingly, available Tunisian data used in the model showed an apparent improvement in physical activity between 1997 and 2009, possibly gaining 45 DPPs. However these results must be interpreted with caution, because different definitions of physical activity were used in two studies.

Increased urbanization normally results in several environmental factors which may discourage participation in physical activity such as: violence, high density traffic, low air quality, pollution and lack of parks, sidewalks and sport/recreation facilities [25].

### Overweight, Obesity and Diabetes

Between 1997 and 2007, BMI increased by an average of 2 kg/m^2^ in men and 1 kg/m^2^ in women and caused over 100 additional CHD deaths. The abdominal obesity prevalence increased in men by 74% and 68% in women between 1997 and 2009. These might explain the discrepancy between the trends in cholesterol and systolic blood pressure in men compared to women. Furthermore, diabetes prevalence, increased from 12% to 17% between 1997 and 2009, this dramatic rise generated approximately 75 additional deaths. One Tunisian national study showed that diabetes prevalence is significantly and independently related to both urban residency and high BMI [26].

The rise in both BMI and Diabetes was responsible for approximately 26% of the increase in CHD deaths. This represents a public health priority. Effective evidence-based interventions exist such as junk-food taxing [27], [28] and should be urgently considered.

The recently published Tunisian formed non-communicable diseases (NCD) strategy adopted an integrated approach and encompassed promotion, prevention and control NCD strategies. To control diabetes and promote physical activity population based multi-sectorial effective evidence-based interventions were clearly indicated such as health promotion, fiscal measures, market control and community participation. However a clear vision and scope of implementation is still evolving [29].

### Smoking

Interestingly, a slight fall in smoking among men resulted in some 55 fewer deaths. Studies conducted over the last 20 years show that the tobacco epidemic in Tunisia is firmly established especially among men [30]. However since 1992 anti-smoking measures started by campaigns of information, enactment of an anti-smoking law, has resulted in a decrease in tobacco sales being observed [31]. Recently in 2010, Tunisia adopted WHO Framework Convention on Tobacco Control, and a strategy was implemented in 2009, with clear goals, and some encouraging results for 2010 [32]. However, the proportion of young and increase in female smokers remains alarming. The consequences of tobacco addiction in Tunisia, in term of prevalence and mortality, will thus be even heavier in the next two decades [31].

### Cardiological Treatments

Modern medical treatments prevented or postponed approximately 445 CHD deaths. The biggest contributions came from treatments delivered in the community for secondary prevention, hypertension and heart failure plus therapies for acute coronary syndromes. The effect of treatment on CHD mortality reduction are thus broadly similar to that reported for Iceland, Sweden and Finland [33] but lower than that reported for North America [34] and Europe [35], [36] in studies using the same methodology.

Despite well established benefits, pharmacological agents continue to be underutilized in Tunisia. As reported by the WHO-PREMISE study, only 6% of eligible CHD patients were receiving statins in 2002 [37]. Also, non compliance rates are alarming, with 63% hypertensive patients not continuing with drug therapies [38], [39].

A UK study suggested that improvements in uptake could make a large impact on the reduction of CHD mortality, which suggested that DPP by current treatments would be double if the uptake levels increase to 80% [40]. Likewise the benefits of medical treatments in Tunisia could be optimized if treatment uptake were improved. However, such policy creates multiple challenges for health providers in term of identifying patients, providing medications and ensuring their long-term compliance.

Data on both patients groups and treatment uptake levels was scarce. The required information in this study was collected directly from the medical-patients records. This revealed some gaps in the NCD health information system. Furthermore treatment uptake levels were not consistent among different hospitals and sometimes physicians, which highlight a lack of standardized health care provision.

### Modeling Strengths and Limitations

The modeling approach used in the study synthesized all the key risk factors and treatment options to help quantify changes in CHD mortality. It usefully provided estimates of the effectiveness and cost-effectiveness for each factor considered [41]. Additionally, the model assessed the potential maximum and minimum plausible effects of these factors using rigorous sensitivity analyses these examined systematically the influence of uncertainties in the assumptions used in the studies.

This modeling approach also has obvious **limitations.** Notably here the extent and quantity of available data on CHD risk factor trends and treatment uptake. However, in general the data used in this model were of good quality. Mortality data were obtained from Ministry of Health death registry [4], [10]. Death registry data was evaluated in previous studies as medium quality based on the WHO criteria. The demographic information was obtained from the censuses data and the risk factor trends were obtained from well designed epidemiological studies and surveys using the WHO STEPS methodology. Treatment uptake and patients groups data were obtained from an extensive hospital based survey conducted in 2009. Data was scarce on treatment uptake. Certain assumptions were needed to fill in the gaps for missing information.

### Public Health Implications

CHD mortality rose by 30 percent between 1997 and 2009 in Tunisia. Almost all of this rise was attributable to increases in major risk factors especially total cholesterol, blood pressure, obesity and diabetes. The prevalence of those risks is high in both men and women and are a concern for both the urban and rural populations. Most hypertensives were not aware of their BP, the treatment rate was low and control of hypertension was poor even on treatment. Our results clearly indicate that risk factors changes currently generate many avoidable CHD deaths.

Conversely, improvements in risk factors in the general population could save many more lives compared to treatments for individual patients. This emphasizes the importance of primary prevention CHD strategies: evidence-based policies which would benefit the whole population.

## Supporting Information

File S1 Appendix S1 and Appendix S2.
(DOCX)
