# Supplementary material for: Analyzing Recent Coronary Heart Disease Mortality Trends in Tunisia between 1997 and 2009
Source: PLoS One. 2013 May 3;8(5):e63202. doi: 10.1371/journal.pone.0063202 (PMC3643918; doi:10.1371/journal.pone.0063202)
Supplement: File S1 — Appendix S1 and Appendix S2. (DOCX) [file pone.0063202.s001.docx]

**Analyzing recent coronary heart disease mortality trends in Tunisia between 1997 and 2009**

O. Saidi *et al.*

# Appendix S1

Contents

APPENDIX S1

[Deaths and population data 4](#_Toc330410213)

[Population Data 4](#_Toc330410214)

[CHD Deaths 4](#_Toc330410215)

[Community CPR 2009 6](#_Toc330410216)

[Hospital CPR 6](#_Toc330410217)

[Proportion of PTCA that are STEMI 6](#_Toc330410218)

[Patient numbers 7](#_Toc330410219)

[Hospital AMI and Unstable angina 7](#_Toc330410220)

[Heart Failure admitted to hospitals 7](#_Toc330410221)

[Secondary prevention post MI (Last 5 years 2009-2005/ Previous 5 years2004-2000) 9](#_Toc330410222)

[Secondary prevention post MI (Last 5 years1992-1997/ Previous 5 years1992-1988) 10](#_Toc330410223)

[Secondary prevention following CABG/PTCA 11](#_Toc330410224)

[Chronic Angina in the community 11](#_Toc330410225)

[CABG numbers for CA (Most recent 5 y) 12](#_Toc330410226)

[CABG numbers for CA (between 10 and 5 y) 12](#_Toc330410227)

[Heart Failure in the community 13](#_Toc330410228)

[Lipid lowering for primary prevention 14](#_Toc330410229)

[Antihypertensive medication numbers 14](#_Toc330410230)

[Risk Factors: 15](#_Toc330410231)

[Smoking prevalence 15](#_Toc330410232)

[SBP 17](#_Toc330410233)

[Cholesterol mean 18](#_Toc330410234)

[BMI mean 18](#_Toc330410235)

[Physical inactivity 19](#_Toc330410236)

[Diabetes prevalence 20](#_Toc330410237)

[Treatment uptake: 21](#_Toc330410238)

[ACUTE MI, Unstable angina 21](#_Toc330410239)

[CPR for acute MI admitted to hospitals 23](#_Toc330410240)

[2' prevention following MI: 2005-2009 23](#_Toc330410241)

[2' prevention following MI: 2000-2004 23](#_Toc330410242)

[2' prevention following MI: 1992-1997 23](#_Toc330410243)

[2' prevention following MI: 1987-1992 24](#_Toc330410244)

[2' prevention following CABG/PTCA 24](#_Toc330410245)

[Chronic angina 26](#_Toc330410246)

[Heart failure in the community 28](#_Toc330410247)

[Lipid lowering for primary prevention 29](#_Toc330410248)

[Antihypertensive medication 30](#_Toc330410249)

[Sources 31](#_Toc330410250)

APPENDIX S2

[Clinical efficacy of interventions: relative risk reductions obtained from meta-analyses, and randomised clinical trials 35](#_Toc330410252)

[Beta coefficients for major risk factors 40](#_Toc330410253)

***Base year : 1997***

***Final Year : 2009***

# Deaths and population data

## Population Data

- **Source**: National Institute of Statistic [1]
- **Quality:** excellent, data available by age (by 5 years) and gender from 1975 to 2010. The older group is up to 80 years and older.

|  | **1997** | **2009** |
| --- | --- | --- |
| **M 25-34** | 732750 | 866176 |
| **M 35-44** | 549000 | 669987 |
| **M 45-54** | 318000 | 571482 |
| **M 55-64** | 260850 | 329595 |
| **M 65-74** | 174450 | 215761 |
| **M 75-84** | 61383 | 94700 |
| **M 85+** | 29067 | 41359 |
| **F 25-34** | 750750 | 907810 |
| **F 35-44** | 546350 | 723103 |
| **F 45-54** | 328950 | 574036 |
| **F 55-64** | 263500 | 347703 |
| **F 65-74** | 201300 | 227208 |
| **F 75-84** | 56617 | 92275 |
| **F 85+** | 28033 | 38802 |
|  |  |  |

## CHD Deaths

- **Sources:** National Institute of Statistic[1]

National Public Health Institute [2]

- 1997 & 2009: Data from the national registry of causes of deaths (National Public Health Institute).
- Before 2001: The national institute of statistics recorded causes of death and they are published in a regular report. However, the causes are classified by groups (Circulatory diseases, respiratory diseases…).
- Since 2001: primary causes of death are coded according to the International Classification of Diseases (ICD10) , using the STIX software.
- We have already analyzed the data for 2001, 2003, 2006, 2009, and estimated the 1997 data using average annual growth rate (2001, 2003).
- **Completeness**
- All deaths are registered,
- Only 50% of primary causes of death are recorded. However, in large cities the percentage is almost 80%.
- Extrapolation: using National Institute of Statistics for mortality data, we applied the % of the recorded causes of death to the number of deaths registered.

|  | **1997** | **2009** |
| --- | --- | --- |
| **M 25-34** | 10 | 11 |
| **M 35-44** | 34 | 82 |
| **M 45-54** | 215 | 261 |
| **M 55-64** | 334 | 426 |
| **M 65-74** | 454 | 645 |
| **M 75-84** | 333 | 713 |
| **M 85+** | 112 | 290 |
| **F 25-34** | 0 | 3 |
| **F 35-44** | 12 | 29 |
| **F 45-54** | 55 | 52 |
| **F 55-64** | 118 | 91 |
| **F 65-74** | 194 | 271 |
| **F 75-84** | 175 | 464 |
| **F 85+** | 60 | 294 |
|  |  |  |

Average (45-54 and 65-74)

## Community CPR 2009

- **Sources:**
- Data from the national registry of emergency: interventions for events occurring in the population.
- Data from some cities (Gafsa and Gabes), we calculated an indicator (number of CPR by 100 000 inhabitants) and we applied this indicator for all the population.

## Hospital CPR

- **Sources:**
- Expert assessment of the occurrence of CPR at the hospitals by hospital level.
- We calculated an indicator: number of CPR for 1000 hospital beds capacity and we applied this rate to the total number of hospitals capacity.

## Proportion of PTCA that are STEMI

- **Source:** Tunisian Epidemiological Study on Acute Coronary Syndrome “ TEPS-ACS 2009/2010” [3]

| **STEMI (ST elevation myocardial infarction)** |  |
| --- | --- |
| AMI (EMERGENCY) admissions (STEMI) | |
| Proportion of PTCA that are STEMI | |
| 0,43 |  |

#

# Patient numbers

## Hospital AMI and Unstable angina

- **Sources**: National Medical Insurance (CNAM) [4]

Tunisian Epidemiological Study on Acute Coronary Syndrome TEPS-ACS 2009/2010 [3]

National survey 2002-2003 [5]

- for 2009, we made an extrapolation using the total number of admissions in cardiology yards ( a total of 23400) and we applied the % (by age group and gender) of the recorded diagnosis in the Tunisian Epidemiological Study on Acute Coronary Syndrome” TEPS-ACS 2009/2010[3] .
- For 2002, we have the total numbers of AMI and unstable angina admitted in all hospitals of Tunisia, and used it to estimate total numbers of AMI and unstable angina admitted on 1997[5].

|  | **Hospital AMI (EMERGENCY) admissions (I21)** | | **Unstable angina** | |
| --- | --- | --- | --- | --- |
|  | **1997** | **2009** | **1997** | **2009** |
| **M 25-34** | 0 | 49 | 23 | 0 |
| **M 35-44** | 128 | 195 | 90 | 331 |
| **M 45-54** | 374 | 927 | 197 | 969 |
| **M 55-64** | 404 | 952 | 181 | 1087 |
| **M 65-74** | 342 | 659 | 190 | 1039 |
| **M 75-84** | 142 | 537 | 69 | 614 |
| **M 85+** | 13 | 146 | 3 | 142 |
| **F 25-34** | 3 | 14 | 5 | 0 |
| **F 35-44** | 21 | 54 | 18 | 71 |
| **F 45-54** | 19 | 195 | 74 | 496 |
| **F 55-64** | 59 | 366 | 152 | 732 |
| **F 65-74** | 131 | 171 | 175 | 425 |
| **F 75-84** | 43 | 171 | 36 | 685 |
| **F 85+** | 16 | 68 | 7 | 165 |
| **Total** | **1695** | **4505** | **1220** | **6755** |

## Heart Failure admitted to hospitals

- **Sources**: National Medical Insurance (CNAM) [4]

National survey 2002-2003 [5]

Regional survey 1997 [6]

- For 2009, the same method was used also to calculate the number chronic heart failure admitted to hospitals from the national survey of 2002.
- We assumed that the number of chronic heart failure admitted to hospitals in 2009 is 1.25 in 2002(Expert opinions)***.
- For 1997, we have the number of chronic heart failure admitted to regional hospitals; we majorated it by 55% [4] to estimate the total admissions in university and private hospitals [6].

| **Heart failure admissions** | | |
| --- | --- | --- |
|  | **1997** | **2009** |
| **M 25-34** | 19 | 29 |
| **M 35-44** | 32 | 49 |
| **M 45-54** | 101 | 153 |
| **M 55-64** | 167 | 254 |
| **M 65-74** | 372 | 566 |
| **M 75-84** | 314 | 478 |
| **M 85+** | 73 | 111 |
| **F 25-34** | 36 | 55 |
| **F 35-44** | 73 | 111 |
| **F 45-54** | 105 | 159 |
| **F 55-64** | 192 | 293 |
| **F 65-74** | 415 | 631 |
| **F 75-84** | 374 | 569 |
| **F 85+** | 105 | 159 |
| **Total** | **2378** | **3614** |

*****:** (Based on the Delphi process, 7 Professors of Cardiology were selected (4 chiefs’ cardiology services in public university hospitals in big Tunis and 3 Professor of Cardiology in 3 private clinics in big Tunis. After collecting responses’ participants by fax and internet, a final meeting was conducted in the Cardiovascular Epidemiology and Prevention Research Laboratory in faculty of medicine of Tunis to validate the results obtained in the model).

## Secondary prevention post MI (Last 5 years 2009-2005/ Previous 5 years2004-2000)

- We used MI admissions 2009, and decreased by 100 yearly (Expert opinions).
- We applied 95% survivals (previous year) and 94 %( the current year) (Expert opinions).

| **Secondary prevention post MI** | **Last 5 years 2009/2004** | **Previous 5 years 2004/2000** |  |
| --- | --- | --- | --- |
|  | **N** | **N** | |
| **M 25-34** | 140 | 118 |  |
| **M 35-44** | 909 | 768 |  |
| **M 45-54** | 2237 | 1891 |  |
| **M 55-64** | 2403 | 2031 |  |
| **M 65-74** | 2735 | 2312 |  |
| **M 75-84** | 1267 | 1071 |  |
| **M 85+** | 79 | 66 |  |
| **F 25-34** | 17 | 15 |  |
| **F 35-44** | 149 | 126 |  |
| **F 45-54** | 367 | 310 |  |
| **F 55-64** | 708 | 598 |  |
| **F 65-74** | 1354 | 1145 |  |
| **F 75-84** | 507 | 428 |  |
| **F 85+** | 166 | 140 |  |
| **Total** | **13038** | **11020** |  |

##

##

## Secondary prevention post MI (Last 5 years1992-1997/ Previous 5 years1992-1988)

- We used 1700 IDM admissions 1997, decreased by 50 yearly (Expert opinions).
- we applied 95% survivals(previous year) and 94%(the current year)
- We used 1200 IDM admissions 1992, and decreased by 20 yearly (Expert opinions).
- we applied 95% survivals(previous year) and 94%(the current year)

| **Secondary prevention post MI** |  |  |
| --- | --- | --- |
|  | **Previous 5 years 1988-1992** | **Last 5 years 1993-1997** |
| M 25-34 | 49 | 105 |
| M 35-44 | 320 | 681 |
| M 45-54 | 789 | 1676 |
| M 55-64 | 847 | 1800 |
| M 65-74 | 964 | 2049 |
| M 75-84 | 447 | 949 |
| M 85+ | 28 | 59 |
| F 25-34 | 6 | 13 |
| F 35-44 | 52 | 111 |
| F 45-54 | 129 | 275 |
| F 55-64 | 250 | 530 |
| F 65-74 | 477 | 1015 |
| F 75-84 | 179 | 380 |
| F 85+ | 59 | 124 |
| **Total** | **4596** | **9768** |

## Secondary prevention following CABG/PTCA

**CABG and PTCA Survival:**

- **Sources**: National Medical Insurance (CNAM) [4]
- Data concerning total number of CABG and PTCA are available for the period 2005 to 2009
- Then with an expert we put an estimated survival rate for each year and calculate the total survivals based on that.
- For the period 2000 to 2004: we made assumptions using trends calculated previously.

| **Secondary prevention following CABG/PTCA (10 years)** | | | | | |
| --- | --- | --- | --- | --- | --- |
| **CABG** | **1997** | **2009** | **PTCA** | **1997** | **2009** |
| M 25-34 | 0 | 30 |  | 0 | 706 |
| M 35-44 | 10 | 30 |  | 20 | 3059 |
| M 45-54 | 100 | 177 |  | 50 | 11061 |
| M 55-64 | 150 | 384 |  | 80 | 9061 |
| M 65-74 | 180 | 295 |  | 90 | 6119 |
| M 75-84 | 40 | 148 |  | 10 | 2706 |
| M 85+ | 0 | 0 |  | 0 | 588 |
| F 25-34 | 0 | 0 |  | 0 | 118 |
| F 35-44 | 2 | 0 |  | 4 | 353 |
| F 45-54 | 4 | 30 |  | 8 | 1647 |
| F 55-64 | 10 | 148 |  | 30 | 3177 |
| F 65-74 | 2 | 30 |  | 4 | 2942 |
| F 75-84 | 2 | 30 |  | 4 | 2118 |
| F 85+ | 0 | 0 |  | 0 | 118 |
| **Total** | **500** | **1300** |  | **300** | **43773** |

## Chronic Angina in the community

- 2009 : we estimated 5% of the Tunisian population aged over 25 years old
- 1997: we estimated 3% of the Tunisian population aged over 25 years old
- After Overlaps & Issues to consider subtract UA

subtract post AMI

subtract half HF

subtract post CABG

subtract post PTCA

| **Chronic Angina in the community (CA)** |  | 1997 |  |  | 2009 |  |
| --- | --- | --- | --- | --- | --- | --- |
|  | **N** | **Overlap correction** | Total angina | **N** | **Overlap correction** | Total angina |
| M 25-34 | 256 | 256 | 512 | -607 | -607 | 1183 |
| M 35-44 | 5709 | 5709 | 6657 | 2582 | 2582 | 15378 |
| M 45-54 | 16877 | 16877 | 19458 | 6040 | 6040 | 44951 |
| M 55-64 | 24339 | 24339 | 27651 | 16285 | 16285 | 63878 |
| M 65-74 | 18831 | 18831 | 24067 | 12379 | 12379 | 55597 |
| M 75-84 | 3170 | 3170 | 6657 | -847 | -847 | 15378 |
| M 85+ | 897 | 897 | 1536 | 267 | 267 | 3549 |
| F 25-34 | 208 | 208 | 512 | 55 | 55 | 1183 |
| F 35-44 | 2377 | 2377 | 3072 | 2148 | 2148 | 7098 |
| F 45-54 | 7076 | 7076 | 8193 | 5994 | 5994 | 18927 |
| F 55-64 | 8659 | 8659 | 10753 | 5742 | 5742 | 24841 |
| F 65-74 | 10540 | 10540 | 14850 | 7268 | 7268 | 34305 |
| F 75-84 | 216 | 216 | 3584 | -2924 | -2924 | 8280 |
| F 85+ | -447 | -447 | 512 | -947 | -947 | 1183 |
| **Total** | **98709** |  |  | **53436** |  |  |

We kept 1% of the Tunisian population aged over 25 years old in 2009 and 2% in 1997.

## CABG numbers for CA (Most recent 5 y)

| **CABG numbers for CA (Most recent 5 y)** | | |
| --- | --- | --- |
|  | **1997** | **2009** |
| M 25-34 | 66 | 131 |
| M 35-44 | 66 | 131 |
| M 45-54 | 393 | 787 |
| M 55-64 | 852 | 1705 |
| M 65-74 | 656 | 1311 |
| M 75-84 | 328 | 656 |
| M 85+ | 0 | 0 |
| F 25-34 | 0 | 0 |
| F 35-44 | 0 | 0 |
| F 45-54 | 66 | 131 |
| F 55-64 | 328 | 656 |
| F 65-74 | 66 | 131 |
| F 75-84 | 66 | 131 |
| F 85+ | 0 | 0 |
| **Total** | **2885** | **5770** |

## CABG numbers for CA (between 10 and 5 y)

| **CABG numbers for CA (between 10 and 5 y)** |  |  |
| --- | --- | --- |
|  | **1997** | 2009 |
| M 25-34 | 41 | 83 |
| M 35-44 | 41 | 83 |
| M 45-54 | 249 | 497 |
| M 55-64 | 539 | 1078 |
| M 65-74 | 414 | 829 |
| M 75-84 | 207 | 414 |
| M 85+ | 0 | 0 |
| F 25-34 | 0 | 0 |
| F 35-44 | 0 | 0 |
| F 45-54 | 41 | 83 |
| F 55-64 | 207 | 414 |
| F 65-74 | 41 | 83 |
| F 75-84 | 41 | 83 |
| F 85+ | 0 | 0 |
| **Total** | **1824** | **3647** |

## Heart Failure in the community

- We assumed that it is 7 times higher than the total Heart failure admitted based on expert opinion.

| **Heart Failure in the community** |  |  |
| --- | --- | --- |
| **2009** | **1997** | **2009** |
| M 25-34 | 135 | 205 |
| M 35-44 | 225 | 341 |
| M 45-54 | 704 | 1069 |
| M 55-64 | 1168 | 1775 |
| M 65-74 | 2605 | 3959 |
| M 75-84 | 2201 | 3344 |
| M 85+ | 509 | 774 |
| F 25-34 | 255 | 387 |
| F 35-44 | 509 | 774 |
| F 45-54 | 734 | 1115 |
| F 55-64 | 1347 | 2048 |
| F 65-74 | 2904 | 4414 |
| F 75-84 | 2620 | 3981 |
| F 85+ | 734 | 1115 |
| **Total** | **16648** | **25298** |

## Lipid lowering for primary prevention

- **Sources**: Tunisian National Survey 2005(TAHINA)[ 7]

Ariana survey 1997[8]

National Institute of Statistic [1]

- We assumed that hypercholesterolemia>= 5.2 mmol/l or treated (%) didn’t change between 2005 and 2009.
- For 2009: We applied hypercholesterolemia>= 5.2 mmol/l or treated (%) [7] to the Tunisian population by age and gender 2009[1].
- For 1997: We applied hypercholesterolemia>= 5.2 mmol/l or treated (%) [8] to the Tunisian population by age and gender 1997[1].
- **Note**: we used Lipid Lowering including statins.

| **Eligible for Statins for primary prevention** | | |
| --- | --- | --- |
|  | **1997** | **2009** |
| M 25-34 | 7328 | 17994 |
| M 35-44 | 57600 | 47593 |
| M 45-54 | 25440 | 71631 |
| M 55-64 | 48306 | 51781 |
| M 65-74 | 20651 | 31535 |
| M 75-84 | 7267 | 14050 |
| M 85+ | 3441 | 6142 |
| F 25-34 | 11261 | 28214 |
| F 35-44 | 76704 | 64363 |
| F 45-54 | 58515 | 98368 |
| F 55-64 | 47909 | 85729 |
| F 65-74 | 26544 | 53230 |
| F 75-84 | 8975 | 21946 |
| F 85+ | 4444 | 9168 |
| **Total** | **404385** | **601744** |

## Antihypertensive medication numbers

- **Sources**: Ariana survey 1997[8]

Ariana survey 2009 [9]

National Institute of Statistic [1]

- From surveys we applied the percentages of antihypertensive medication uptake to the Tunisian population by age and gender 1997, 2009.

| **Eligible for Antihypertensive medication** | | |
| --- | --- | --- |
|  | **1997** | **2009** |
| M 25-34 | 43965 | 71975 |
| M 35-44 | 134878 | 110638 |
| M 45-54 | 119772 | 156020 |
| M 55-64 | 140268 | 139986 |
| M 65-74 | 93808 | 112733 |
| M 75-84 | 33008 | 54161 |
| M 85+ | 15630 | 25824 |
| F 25-34 | 75075 | 75239 |
| F 35-44 | 157998 | 108071 |
| F 45-54 | 177856 | 201162 |
| F 55-64 | 167007 | 195824 |
| F 65-74 | 106130 | 154692 |
| F 75-84 | 35884 | 63778 |
| F 85+ | 17768 | 26642 |
| **Total** | **1319047** | **1496744** |

# Risk Factors:

## Smoking prevalence

- **Sources:**

Tunisian National survey, INSP 1996-1997[10]

Tunisian National Survey 2005(TAHINA) [7]

- The smokers category included current smokers who were smoking daily and had been doing so for at least the previous month, and ex-smokers who had smoked regularly for a year but were no longer smoking.
- We assumed that Smoking prevalence didn’t change between 2005 and 2009.

**Note:** For younger and older age groups, some groups are very small or null, so for these groups we assume that the risk factor profile is similar to the next younger group and the previous older age groups and we validated with experts opinions.

| **Smoking** |  |  |  |  |
| --- | --- | --- | --- | --- |
| **Prevalence** |  |  |  |  |
|  | **1997** |  | **2009** | Change |
| M 25-34 | 0,588 |  | 0,477 | -0,11 |
| M 35-44 | 0,497 |  | 0,638 | 0,14 |
| M 45-54 | 0,434 |  | 0,483 | 0,05 |
| M 55-64 | 0,334 |  | 0,404 | 0,07 |
| M 65-74 | 0,334 |  | 0,247 | -0,09 |
| M 75-84 | 0,343 |  | 0,250 | -0,09 |
| M 85+ | 0,300 |  | 0,250 | -0,05 |
| F 25-34 | 0,025 |  | 0,033 | 0,01 |
| F 35-44 | 0,019 |  | 0,025 | 0,01 |
| F 45-54 | 0,020 |  | 0,020 | 0,00 |
| F 55-64 | 0,020 |  | 0,020 | 0,00 |
| F 65-74 | 0,014 |  | 0,020 | 0,01 |
| F 75-84 | 0,010 |  | 0,014 | 0,00 |
| F 85+ | 0,000 |  | 0,010 | 0,01 |

## SBP

- **Sources:**

Tunisian National Survey 2005(TAHINA) [7]

Ariana survey 1997[8]

Ariana survey 2009[9]

- The comparison of Tunisian National Survey 2005 and Ariana survey 2009 did not show any difference concerning risk factors so Ariana survey 2009 is a representative sample.

| **SBP** | |  |  |  |
| --- | --- | --- | --- | --- |
|  | **1997** |  | **2009** |  |
| M 25-34 | 124,2 |  | 127,6 | 3,49 |
| M 35-44 | 126,2 |  | 129,3 | 3,12 |
| M 45-54 | 129,9 |  | 134,4 | 4,49 |
| M 55-64 | 136,8 |  | 143,9 | 7,11 |
| M 65-74 | 142,3 |  | 146,2 | 3,88 |
| M 75-84 | 140,3 |  | 142,3 | 1,94 |
| M 85+ | 136,0 |  | 138,0 | 2,00 |
| F 25-34 | 122,3 |  | 120,6 | -1,72 |
| F 35-44 | 125,2 |  | 123,3 | -1,87 |
| F 45-54 | 136,2 |  | 132,2 | -4,03 |
| F 55-64 | 142,9 |  | 138,9 | -4,02 |
| F 65-74 | 147,9 |  | 144,5 | -3,46 |
| F 75-84 | 146,7 |  | 146,3 | -0,45 |
| F 85+ | 147,8 |  | 146,3 | -1,55 |

## Cholesterol mean

- **Sources:**

Tunisian National Survey 2005(TAHINA) [7]

Ariana survey 1997[8]

- We assumed that Cholesterol mean didn’t change between 2005 and 2009.

| **Cholesterol** |  |  |  |  |  |
| --- | --- | --- | --- | --- | --- |
|  | **1997** |  | **2009** |  |  |
| M 25-34 | 4,78 |  | 4,80 | 0,02 | Rise |
| M 35-44 | 4,78 |  | 4,80 | 0,02 | Rise |
| M 45-54 | 4,76 |  | 4,91 | 0,15 | Rise |
| M 55-64 | 5,18 |  | 5,09 | -0,09 |  |
| M 65-74 | 5,08 |  | 5,32 | 0,24 | Rise |
| M 75-84 | 5,08 |  | 5,32 | 0,24 | Rise |
| M 85+ | 5,08 |  | 5,32 | 0,24 | Rise |
| F 25-34 | *4,94* |  | *4,30* | -0,63 | FALL |
| F 35-44 | *4,94* |  | *4,30* | -0,63 | FALL |
| F 45-54 | *5,16* |  | *4,54* | -0,62 | FALL |
| F 55-64 | *5,25* |  | *4,67* | -0,58 | FALL |
| F 65-74 | *5,39* |  | *4,75* | -0,65 | FALL |
| F 75-84 | *5,39* |  | *4,75* | -0,65 | FALL |
| F 85+ | *5,39* |  | *4,75* | -0,65 | FALL |

## BMI mean

- **Sources:**

Ariana survey 1997[8]

Ariana survey 2009[9]

- We assumed that Ariana survey 1997 and 2009 are representative samples.

|  |  |  |  |  |
| --- | --- | --- | --- | --- |
| **BMI** | **1997** |  | **2009** |  |
| M 25-34 | 24,32 |  | 25,32 | 1,00 |
| M 35-44 | 25,02 |  | 26,89 | 1,87 |
| M 45-54 | 25,29 |  | 28,91 | 3,62 |
| M 55-64 | 25,09 |  | 27,51 | 2,42 |
| M 65-74 | 25,14 |  | 26,63 | 1,50 |
| M 75-84 | 24,13 |  | 25,03 | 0,90 |
| M 85+ | 24,01 |  | 24,03 | 0,02 |
| F 25-34 | 25,96 |  | 25,86 | -0,10 |
| F 35-44 | 27,02 |  | 28,75 | 1,73 |
| F 45-54 | 27,95 |  | 28,73 | 0,78 |
| F 55-64 | 27,83 |  | 29,43 | 1,59 |
| F 65-74 | 28,96 |  | 30,91 | 1,95 |
| F 75-84 | 28,18 |  | 30,10 | 1,92 |
| F 85+ | 28,16 |  | 29,86 | 1,70 |

## Physical inactivity

- **Sources:**

Ariana survey 1997[8]

Ariana survey 2009[9]

- We assumed that Ariana survey 1997 and 2009 are representative samples.
- We included data about physical activities, but it carries some risks due to the different definition of physical activity between 1997 (WHO definition) and 2009(IPAQ definition).

|  | **Tunisia** |  | **Tunisia** |  |
| --- | --- | --- | --- | --- |
| **Physical inactivity** | **1997** |  | **2009** |  |
| M 25-34 | 0.945 |  | 0.779 | -0.17 |
| M 35-44 | 0.975 |  | 0.797 | -0.18 |
| M 45-54 | 0.986 |  | 0.800 | -0.19 |
| M 55-64 | 0.991 |  | 0.865 | -0.13 |
| M 65-74 | 1.000 |  | 0.877 | -0.12 |
| M 75-84 | 1.000 |  | 1.000 | 0.00 |
| M 85+ | 1.000 |  | 1.000 | 0.00 |
| F 25-34 | 0.987 |  | 0.878 | -0.11 |
| F 35-44 | 0.997 |  | 0.842 | -0.15 |
| F 45-54 | 1.000 |  | 0.875 | -0.13 |
| F 55-64 | 1.000 |  | 0.936 | -0.06 |
| F 65-74 | 1.000 |  | 0.933 | -0.07 |
| F 75-84 | 1.000 |  | 1.000 | 0.00 |
| F 85+ | 1.000 |  | 1.000 | 0.00 |

## Diabetes prevalence

- **Sources:**

Tunisian National Nutrition Survey 1996/97[11]

Tunisian National Survey 2005(TAHINA) [7]

- Type 2 diabetes cases were then defined as subjects with measured FG>=6.1 mmol/l or having a previously diagnosed diabetes.
- We assumed that Diabetes prevalence didn’t change between 2005 and 2009.

| **Diabetes** | **Country Name** |  | **Country Name** |  |
| --- | --- | --- | --- | --- |
|  | **1997** |  | **2009** |  |
| M 25-34 | 0,080 |  | 0,053 | -0,03 |
| M 35-44 | 0,086 |  | 0,107 | 0,02 |
| M 45-54 | 0,156 |  | 0,180 | 0,02 |
| M 55-64 | 0,184 |  | 0,223 | 0,04 |
| M 65-74 | 0,153 |  | 0,220 | 0,07 |
| M 75-84 | 0,180 |  | 0,220 | 0,04 |
| M 85+ | 0,180 |  | 0,220 | 0,04 |
| F 25-34 | 0,022 |  | 0,035 | 0,01 |
| F 35-44 | 0,068 |  | 0,071 | 0,00 |
| F 45-54 | 0,086 |  | 0,149 | 0,06 |
| F 55-64 | 0,136 |  | 0,227 | 0,09 |
| F 65-74 | 0,210 |  | 0,264 | 0,05 |
| F 75-84 | 0,222 |  | 0,264 | 0,04 |
| F 85+ | 0,264 |  | 0,264 | 0,00 |

# Treatment uptake:

## ACUTE MI, Unstable angina

- **Sources:**

Survey AMI 2002 [12]

TEPS-ACS- “Tunisian Epidemiological Study on ACS “2009- 2010[3]

- For 1997: We used Survey AMI 2002 to assume treatment uptake 1997.

| **ACUTE MI 2009** | Aspirin | ACE inhibitors | Beta-blockers | CABG | PTCA (STEMI) | Rehabilitation | CRP in community | CPR in hospital | Thrombolysis |
| --- | --- | --- | --- | --- | --- | --- | --- | --- | --- |
| M 25-34 | 0,933 | 0,733 | 0,800 | 0,067 | 0,500 | 0 | 0,05 | 0,5 | 0,333 |
| M 35-44 | 1,000 | 0,829 | 0,829 | 0,026 | 0,643 | 0 | 0,05 | 0,5 | 0,317 |
| M 45-54 | 0,953 | 0,688 | 0,740 | 0,032 | 0,476 | 0 | 0,05 | 0,5 | 0,255 |
| M 55-64 | 0,928 | 0,692 | 0,733 | 0,054 | 0,427 | 0 | 0,05 | 0,5 | 0,237 |
| M 65-74 | 0,971 | 0,745 | 0,591 | 0,053 | 0,400 | 0 | 0,05 | 0,5 | 0,181 |
| M 75-84 | 0,929 | 0,690 | 0,583 | 0,036 | 0,306 | 0 | 0,05 | 0,5 | 0,116 |
| M 85+ | 0,833 | 0,667 | 0,444 | 0,000 | 0,571 | 0 | 0,05 | 0,5 | 0,056 |
| F 25-34 | 1,000 | 0,500 | 0,500 | 0,000 | 1,000 | 0 | 0,05 | 0,5 | 0,000 |
| F 35-44 | 1,000 | 0,750 | 1,000 | 0,000 | 0,000 | 0 | 0,05 | 0,5 | 0,250 |
| F 45-54 | 0,963 | 0,852 | 0,741 | 0,038 | 0,692 | 0 | 0,05 | 0,5 | 0,148 |
| F 55-64 | 0,969 | 0,778 | 0,683 | 0,078 | 0,472 | 0 | 0,05 | 0,5 | 0,281 |
| F 65-74 | 0,904 | 0,685 | 0,658 | 0,000 | 0,235 | 0 | 0,05 | 0,5 | 0,122 |
| F 75-84 | 0,982 | 0,509 | 0,509 | 0,018 | 0,368 | 0 | 0,05 | 0,5 | 0,070 |
| F 85+ | 1,000 | 1,000 | 0,667 | 0,000 | 0,200 | 0 | 0,05 | 0,5 | 0,167 |

| **ACUTE MI 1997** | Aspirin | ACE inhibitors | Beta-blockers | CABG | PTCA (STEMI) | Rehabilitation | CRP in community | CPR in hospital | Thrombolysis |
| --- | --- | --- | --- | --- | --- | --- | --- | --- | --- |
| M 25-34 | 0,000 | 0,000 | 0,000 | 0,067 | 0,000 | 0,000 | 0,000 | 0,000 | 0,000 |
| M 35-44 | 0,656 | 0,484 | 0,935 | 0,026 | 0,000 | 0,000 | 0,000 | 0,000 | 0,115 |
| M 45-54 | 0,702 | 0,493 | 0,921 | 0,032 | 0,000 | 0,000 | 0,000 | 0,000 | 0,146 |
| M 55-64 | 0,641 | 0,536 | 0,885 | 0,054 | 0,000 | 0,000 | 0,000 | 0,000 | 0,127 |
| M 65-74 | 0,645 | 0,480 | 0,798 | 0,053 | 0,000 | 0,000 | 0,000 | 0,000 | 0,125 |
| M 75-84 | 0,623 | 0,509 | 0,712 | 0,036 | 0,000 | 0,000 | 0,000 | 0,000 | 0,108 |
| M 85+ | 0,600 | 0,300 | 0,600 | 0,000 | 0,000 | 0,000 | 0,000 | 0,000 | 0,050 |
| F 25-34 | 0,000 | 0,000 | 1,000 | 0,000 | 0,000 | 0,000 | 0,000 | 0,000 | 0,000 |
| F 35-44 | 0,750 | 0,563 | 1,000 | 0,000 | 0,000 | 0,000 | 0,000 | 0,000 | 0,125 |
| F 45-54 | 0,750 | 0,536 | 0,857 | 0,038 | 0,000 | 0,000 | 0,000 | 0,000 | 0,036 |
| F 55-64 | 0,682 | 0,614 | 0,864 | 0,078 | 0,000 | 0,000 | 0,000 | 0,000 | 0,068 |
| F 65-74 | 0,704 | 0,551 | 0,918 | 0,000 | 0,000 | 0,000 | 0,000 | 0,000 | 0,138 |
| F 75-84 | 0,703 | 0,375 | 0,813 | 0,018 | 0,000 | 0,000 | 0,000 | 0,000 | 0,063 |
| F 85+ | 0,750 | 0,625 | 0,833 | 0,000 | 0,000 | 0,000 | 0,000 | 0,000 | 0,000 |

|  | **1997** | | | | | **2009** | | | | |
| --- | --- | --- | --- | --- | --- | --- | --- | --- | --- | --- |
| **Unstable angina** | Aspirin | Aspirin & Heparin | PG IIA/IIB | CABG | PTCA (STEMI) | Aspirin | Aspirin & Heparin | PG IIA/IIB | CABG | PTCA |
| M 25-34 | 0,000 | 0,000 | 0,000 | 0,000 | 0,000 | 0,150 | 0,800 | 0,000 | 0,000 | 0,050 |
| M 35-44 | 0,700 | 0,000 | 0,000 | 0,000 | 0,000 | 0,150 | 0,800 | 0,000 | 0,000 | 0,050 |
| M 45-54 | 0,682 | 0,000 | 0,000 | 0,105 | 0,000 | 0,150 | 0,800 | 0,026 | 0,070 | 0,050 |
| M 55-64 | 0,700 | 0,000 | 0,000 | 0,000 | 0,000 | 0,150 | 0,800 | 0,048 | 0,000 | 0,050 |
| M 65-74 | 0,646 | 0,000 | 0,000 | 0,273 | 0,000 | 0,307 | 0,643 | 0,000 | 0,182 | 0,050 |
| M 75-84 | 0,700 | 0,000 | 0,000 | 0,000 | 0,000 | 0,350 | 0,600 | 0,000 | 0,000 | 0,050 |
| M 85+ | 0,700 | 0,000 | 0,000 | 0,060 | 0,000 | 0,950 | 0,000 | 0,000 | 0,040 | 0,050 |
| F 25-34 | 0,000 | 0,000 | 0,000 | 0,000 | 0,000 | 0,950 | 0,000 | 0,000 | 0,000 | 0,050 |
| F 35-44 | 0,525 | 0,000 | 0,000 | 0,000 | 0,000 | 0,450 | 0,500 | 0,000 | 0,000 | 0,050 |
| F 45-54 | 0,700 | 0,000 | 0,000 | 0,000 | 0,000 | 0,117 | 0,833 | 0,000 | 0,000 | 0,050 |
| F 55-64 | 0,672 | 0,000 | 0,000 | 0,000 | 0,000 | 0,117 | 0,833 | 0,000 | 0,000 | 0,050 |
| F 65-74 | 0,700 | 0,000 | 0,000 | 0,065 | 0,000 | 0,533 | 0,417 | 0,000 | 0,043 | 0,050 |
| F 75-84 | 0,630 | 0,000 | 0,000 | 0,000 | 0,000 | 0,533 | 0,417 | 0,000 | 0,000 | 0,050 |
| F 85+ | 0,000 | 0,000 | 0,000 | 0,000 | 0,000 | 1,000 | 0,000 | 0,000 | 0,000 | 0,050 |

## CPR for acute MI admitted to hospitals

- Expert opinions suggest 5% in community and 50% in hospitals.

## 2' prevention following MI: 2005-2009

- **Source:**

PREMISE II: 2010 [14]

- We assumed that the % of treatment between 2005 and 2009 didn’t change.

| **2' prevention following MI : 2005-2009** | Statins | Aspirin | Warfarin | ACE inhibitors | Beta-blockers |
| --- | --- | --- | --- | --- | --- |
| M 25-34 | 0,64 | 0,91 | 0,00 | 0,00 | 0,63 |
| M 35-44 | 0,64 | 0,91 | 0,00 | 0,75 | 0,63 |
| M 45-54 | 0,64 | 0,91 | 0,00 | 0,64 | 0,63 |
| M 55-64 | 0,78 | 0,96 | 0,00 | 0,89 | 0,63 |
| M 65-74 | 0,77 | 0,96 | 0,00 | 0,69 | 0,63 |
| M 75-84 | 0,82 | 0,91 | 0,00 | 0,64 | 0,63 |
| M 85+ | 0,00 | 0,91 | 0,00 | 0,33 | 0,33 |
| F 25-34 | 1,00 | 0,91 | 0,00 | 0,75 | 1,00 |
| F 35-44 | 0,75 | 0,91 | 0,00 | 0,75 | 0,50 |
| F 45-54 | 0,75 | 0,91 | 0,00 | 0,75 | 0,50 |
| F 55-64 | 0,73 | 0,80 | 0,00 | 0,73 | 0,50 |
| F 65-74 | 0,59 | 0,93 | 0,00 | 0,64 | 0,50 |
| F 75-84 | 0,50 | 0,90 | 0,00 | 0,64 | 0,50 |
| F 85+ | 0,00 | 0,40 | 0,00 | 0,34 | 0,40 |

## 2' prevention following MI: 2000-2004

- **Source:**

PREMISE I: 2002 [13]

- We assumed that the % of treatment between 2000 and 2004 didn’t change.

| **2' prevention following MI : 2000-2004** | Statins | Aspirin | Warfarin | ACE inhibitors | Beta-blockers | Rehabilitation |
| --- | --- | --- | --- | --- | --- | --- |
| M 25-34 | 0,32 | 0,45 | 0,00 | 0,00 | 0,31 | 0,00 |
| M 35-44 | 0,32 | 0,45 | 0,00 | 0,38 | 0,31 | 0,00 |
| M 45-54 | 0,32 | 0,45 | 0,00 | 0,32 | 0,31 | 0,00 |
| M 55-64 | 0,39 | 0,48 | 0,00 | 0,44 | 0,31 | 0,00 |
| M 65-74 | 0,38 | 0,48 | 0,00 | 0,35 | 0,31 | 0,00 |
| M 75-84 | 0,41 | 0,45 | 0,00 | 0,32 | 0,31 | 0,00 |
| M 85+ | 0,00 | 0,45 | 0,00 | 0,17 | 0,17 | 0,00 |
| F 25-34 | 0,50 | 0,45 | 0,00 | 0,38 | 0,50 | 0,00 |
| F 35-44 | 0,38 | 0,45 | 0,00 | 0,38 | 0,25 | 0,00 |
| F 45-54 | 0,38 | 0,45 | 0,00 | 0,38 | 0,25 | 0,00 |
| F 55-64 | 0,37 | 0,40 | 0,00 | 0,37 | 0,25 | 0,00 |
| F 65-74 | 0,29 | 0,46 | 0,00 | 0,32 | 0,25 | 0,00 |
| F 75-84 | 0,25 | 0,45 | 0,00 | 0,32 | 0,25 | 0,00 |
| F 85+ | 0,00 | 0,20 | 0,00 | 0,17 | 0,20 | 0,00 |

## 2' prevention following MI: 1992-1997

- **Source:**

PREMISE I: 2002 [13]

- We assumed that the % of treatment between 1992 and 1997 is the half of 2002 (Expert opinion).

| **2' prevention following MI : 1992-1997** | Statins | Aspirin | Warfarin | ACE inhibitors | Beta-blockers | Rehabilitation |
| --- | --- | --- | --- | --- | --- | --- |
| M 25-34 | 0,000 | 0,000 | 0,000 | 0,00 | 0,31 | 0,000 |
| M 35-44 | 0,000 | 0,350 | 0,000 | 0,17 | 0,31 | 0,000 |
| M 45-54 | 0,000 | 0,341 | 0,000 | 0,18 | 0,31 | 0,000 |
| M 55-64 | 0,000 | 0,350 | 0,000 | 0,24 | 0,31 | 0,000 |
| M 65-74 | 0,000 | 0,323 | 0,000 | 0,39 | 0,31 | 0,000 |
| M 75-84 | 0,000 | 0,350 | 0,000 | 0,17 | 0,31 | 0,000 |
| M 85+ | 0,000 | 0,350 | 0,000 | 0,00 | 0,17 | 0,000 |
| F 25-34 | 0,000 | 0,000 | 0,000 | 0,00 | 0,50 | 0,000 |
| F 35-44 | 0,000 | 0,263 | 0,000 | 0,00 | 0,25 | 0,000 |
| F 45-54 | 0,000 | 0,350 | 0,000 | 0,33 | 0,25 | 0,000 |
| F 55-64 | 0,000 | 0,336 | 0,000 | 0,13 | 0,25 | 0,000 |
| F 65-74 | 0,000 | 0,350 | 0,000 | 0,33 | 0,25 | 0,000 |
| F 75-84 | 0,000 | 0,315 | 0,000 | 0,00 | 0,25 | 0,000 |
| F 85+ | 0,000 | 0,000 | 0,000 | 0,00 | 0,20 | 0,000 |

## 2' prevention following MI: 1987-1992

- **Source:**

PREMISE I: 2002[13]

- We assumed that the % of treatment between 1992 and 1997 is the half of 1997(Expert opinion).

| **2' prevention following MI (ADG3b): 1987-1992** | Statins | Aspirin | Warfarin | ACE inhibitors | Beta-blockers | Rehabilitation |
| --- | --- | --- | --- | --- | --- | --- |
| M 25-34 | 0,000 | 0,000 | 0,000 | 0,000 | 0,16 | 0,000 |
| M 35-44 | 0,000 | 0,350 | 0,000 | 0,000 | 0,16 | 0,000 |
| M 45-54 | 0,000 | 0,341 | 0,000 | 0,000 | 0,16 | 0,000 |
| M 55-64 | 0,000 | 0,350 | 0,000 | 0,000 | 0,16 | 0,000 |
| M 65-74 | 0,000 | 0,323 | 0,000 | 0,000 | 0,16 | 0,000 |
| M 75-84 | 0,000 | 0,350 | 0,000 | 0,000 | 0,16 | 0,000 |
| M 85+ | 0,000 | 0,350 | 0,000 | 0,000 | 0,08 | 0,000 |
| F 25-34 | 0,000 | 0,000 | 0,000 | 0,000 | 0,25 | 0,000 |
| F 35-44 | 0,000 | 0,263 | 0,000 | 0,000 | 0,13 | 0,000 |
| F 45-54 | 0,000 | 0,350 | 0,000 | 0,000 | 0,13 | 0,000 |
| F 55-64 | 0,000 | 0,336 | 0,000 | 0,000 | 0,13 | 0,000 |
| F 65-74 | 0,000 | 0,350 | 0,000 | 0,000 | 0,13 | 0,000 |
| F 75-84 | 0,000 | 0,315 | 0,000 | 0,000 | 0,13 | 0,000 |
| F 85+ | 0,000 | 0,000 | 0,000 | 0,000 | 0,10 | 0,000 |

## 2' prevention following CABG/PTCA

- **Sources:**

PREMISE I: 2002[13]

PREMISE II: 2010[14]

- For 1997: We used **PREMISE I 2002to** assume treatment uptake 1997.

| **2' prevention following CABG/PTCA (DG4) 2009** | Statins | Aspirin | Warfarin | ACE inhibitors | Beta-blockers | Rehabilitation |
| --- | --- | --- | --- | --- | --- | --- |
| M 25-34 | 0,00 | 0,91 | 0,00 | 0,00 | 0,00 | 0,00 |
| M 35-44 | 0,64 | 0,91 | 0,00 | 0,50 | 0,40 | 0,00 |
| M 45-54 | 0,64 | 0,91 | 0,00 | 0,50 | 0,40 | 0,00 |
| M 55-64 | 0,83 | 0,96 | 0,00 | 0,50 | 0,40 | 0,00 |
| M 65-74 | 0,81 | 0,96 | 0,00 | 0,50 | 0,40 | 0,00 |
| M 75-84 | 0,75 | 0,96 | 0,00 | 0,50 | 0,40 | 0,00 |
| M 85+ | 0,00 | 0,96 | 0,00 | 0,50 | 0,40 | 0,00 |
| F 25-34 | 0,00 | 0,96 | 0,00 | 0,50 | 0,40 | 0,00 |
| F 35-44 | 0,00 | 0,96 | 0,00 | 0,50 | 0,40 | 0,00 |
| F 45-54 | 0,62 | 0,96 | 0,00 | 0,50 | 0,40 | 0,00 |
| F 55-64 | 0,62 | 0,96 | 0,08 | 0,50 | 0,40 | 0,00 |
| F 65-74 | 0,62 | 0,96 | 0,00 | 0,50 | 0,40 | 0,00 |
| F 75-84 | 0,32 | 0,96 | 0,00 | 0,50 | 0,40 | 0,00 |
| F 85+ | 0,00 | 0,00 | 0,00 | 0,00 | 0,00 | 0,00 |

| **2' prevention following CABG/PTCA (ADG4) 1997** | Statins | Aspirin | Warfarin | ACE inhibitors | Beta-blockers | Rehabilitation |
| --- | --- | --- | --- | --- | --- | --- |
| M 25-34 | 0,000 | 0,000 | 0,000 | 0,000 | 0,000 | 0,000 |
| M 35-44 | 0,000 | 1,000 | 0,000 | 0,333 | 1,000 | 0,000 |
| M 45-54 | 0,000 | 0,905 | 0,000 | 0,350 | 0,750 | 0,000 |
| M 55-64 | 0,000 | 1,000 | 0,000 | 0,476 | 0,682 | 0,000 |
| M 65-74 | 0,000 | 0,842 | 0,000 | 0,778 | 0,684 | 0,000 |
| M 75-84 | 0,000 | 0,667 | 0,000 | 0,333 | 1,000 | 0,000 |
| M 85+ | 0,000 | 0,000 | 0,000 | 0,000 | 0,000 | 0,000 |
| F 25-34 | 0,000 | 0,000 | 0,000 | 0,000 | 0,000 | 0,000 |
| F 35-44 | 0,000 | 0,000 | 0,000 | 0,000 | 0,000 | 0,000 |
| F 45-54 | 0,000 | 1,000 | 0,000 | 0,000 | 0,010 | 0,000 |
| F 55-64 | 0,000 | 0,750 | 0,000 | 0,000 | 0,008 | 0,000 |
| F 65-74 | 0,000 | 0,750 | 0,000 | 0,000 | 0,008 | 0,000 |
| F 75-84 | 0,000 | 0,000 | 0,000 | 0,000 | 0,000 | 0,000 |
| F 85+ | 0,000 | 0,000 | 0,000 | 0,000 | 0,000 | 0,000 |

## Chronic angina

- **Sources:**

PREMISE I: 2002[13]

PREMISE II: 2010[14]

For 2009:

- we used PREMISE II for Aspirin and Statins
- We assumed that CABG for chronic angina is 4 times higher than CABG for unstable angina based on expert opinion.

For 1997:

- We assumed that Aspirin didn’t change between 1997 and 2009
- We assumed that Statins and PTCA are nulls
- we used PREMISE I for CABG

|  | **1997** | | | | **2009** | | | |
| --- | --- | --- | --- | --- | --- | --- | --- | --- |
| **Chronic angina (DG5)** | Statins | Aspirin | CABG | PTCA | Statins | Aspirin | CABG | PTCA |
| M 25-34 | 0,000 | 1,00 | 0,000 | 0,000 | 0,50 | 0,50 | 0,00 | 0,00 |
| M 35-44 | 0,000 | 1,00 | 0,000 | 0,000 | 0,50 | 0,50 | 0,00 | 1,00 |
| M 45-54 | 0,000 | 0,78 | 0,279 | 0,000 | 0,78 | 0,78 | 0,28 | 0,41 |
| M 55-64 | 0,000 | 0,89 | 0,000 | 0,000 | 0,72 | 0,89 | 0,00 | 0,20 |
| M 65-74 | 0,000 | 0,90 | 0,727 | 0,000 | 0,50 | 0,90 | 0,73 | 0,75 |
| M 75-84 | 0,000 | 0,80 | 0,000 | 0,000 | 0,25 | 0,80 | 0,00 | 0,73 |
| M 85+ | 0,000 | 0,60 | 0,161 | 0,000 | 0,25 | 0,80 | 0,16 | 0,00 |
| F 25-34 | 0,000 | 1,00 | 0,000 | 0,000 | 0,50 | 0,90 | 0,00 | 0,00 |
| F 35-44 | 0,000 | 1,00 | 0,000 | 0,000 | 0,50 | 0,90 | 0,00 | 0,67 |
| F 45-54 | 0,000 | 0,75 | 0,000 | 0,000 | 0,50 | 0,75 | 0,00 | 0,43 |
| F 55-64 | 0,000 | 0,67 | 0,000 | 0,000 | 0,50 | 0,67 | 0,00 | 0,27 |
| F 65-74 | 0,000 | 0,63 | 0,174 | 0,000 | 0,50 | 0,63 | 0,17 | 0,36 |
| F 75-84 | 0,000 | 0,70 | 0,000 | 0,000 | 0,25 | 0,50 | 0,00 | 0,44 |
| F 85+ | 0,000 | 0,50 | 0,000 | 0,000 | 0,01 | 0,40 | 0,00 | 0,00 |

**Heart failure with hospital admission**

- For 2009: We assumed that Aspirin for Heart failure with hospital admission is the same of Aspirin for MI based on expert opinion.
- For 2009: Expert opinions assumed 0% for statins, 20% ACE inhibitors, 50 % Beta-blockers, 70% Spironolactone.
- For 1997: We assumed that Aspirin and Beta-blockers for Heart failure with hospital admission are the same for MI based on expert opinion.
- For 1997: Expert opinions assumed 0% for statins, 0% ACE inhibitors, 60% Spironolactone.

|  | **1997** | | | | | **2009** | | | | | | | | |
| --- | --- | --- | --- | --- | --- | --- | --- | --- | --- | --- | --- | --- | --- | --- |
| **Heart failure with hospital admission** | Statins | Aspirin | ACE inhibitors | Beta-blockers | Spironolactone | Statins | Aspirin | | ACE inhibitors | | Beta-blockers | | Spironolactone | |
| M 25-34 | 0,000 | 0,000 | 0,000 | 0,000 | 0,600 | 0,000 | | 0,933 | | 0,20 | | 0,50 | | 0,25 |
| M 35-44 | 0,000 | 0,656 | 0,000 | 0,935 | 0,600 | 0,000 | 1,000 | | 0,20 | | 0,50 | | 0,25 | |
| M 45-54 | 0,000 | 0,702 | 0,000 | 0,921 | 0,600 | 0,000 | 0,953 | | 0,20 | | 0,50 | | 0,25 | |
| M 55-64 | 0,000 | 0,641 | 0,000 | 0,885 | 0,600 | 0,000 | 0,928 | | 0,20 | | 0,50 | | 0,25 | |
| M 65-74 | 0,000 | 0,645 | 0,000 | 0,798 | 0,600 | 0,000 | 0,971 | | 0,20 | | 0,50 | | 0,25 | |
| M 75-84 | 0,000 | 0,623 | 0,000 | 0,712 | 0,600 | 0,000 | 0,929 | | 0,20 | | 0,50 | | 0,25 | |
| M 85+ | 0,000 | 0,600 | 0,000 | 0,600 | 0,600 | 0,000 | 0,833 | | 0,20 | | 0,50 | | 0,25 | |
| F 25-34 | 0,000 | 0,000 | 0,000 | 1,000 | 0,600 | 0,000 | 1,000 | | 0,20 | | 0,50 | | 0,25 | |
| F 35-44 | 0,000 | 0,750 | 0,000 | 1,000 | 0,600 | 0,000 | 1,000 | | 0,20 | | 0,50 | | 0,25 | |
| F 45-54 | 0,000 | 0,750 | 0,000 | 0,857 | 0,600 | 0,000 | 0,963 | | 0,20 | | 0,50 | | 0,25 | |
| F 55-64 | 0,000 | 0,682 | 0,000 | 0,864 | 0,600 | 0,000 | 0,969 | | 0,20 | | 0,50 | | 0,25 | |
| F 65-74 | 0,000 | 0,704 | 0,000 | 0,918 | 0,600 | 0,000 | 0,904 | | 0,20 | | 0,50 | | 0,25 | |
| F 75-84 | 0,000 | 0,703 | 0,000 | 0,813 | 0,600 | 0,000 | 0,982 | | 0,20 | | 0,50 | | 0,25 | |
| F 85+ | 0,000 | 0,750 | 0,000 | 0,833 | 0,600 | 0,000 | 1,000 | | 0,20 | | 0,50 | | 0,25 | |

## Heart failure in the community

- **Sources:**

PREMISE I: 2002[13]

PREMISE II: 2010 [14]

- For 1997: We used PREMISE I 2002 to assume treatment uptake 1997.

|  | **1997** | | | | | **2009** | | | | |
| --- | --- | --- | --- | --- | --- | --- | --- | --- | --- | --- |
| **Heart failure in the community** | Statins | Aspirin | ACE inhibitors | Beta-blockers | Spironolactone | Statins | Aspirin | ACE inhibitors | Beta-blockers | Spironolactone |
| M 25-34 | 0,000 | 0,0000 | 0,100 | 0,250 | 0,08333 | 0,000 | 0,000 | 0,000 | 0,000 | 0,000 |
| M 35-44 | 0,000 | 0,0000 | 0,100 | 0,250 | 0,08333 | 0,000 | 0,500 | 0,167 | 0,500 | 0,000 |
| M 45-54 | 0,500 | 1,0000 | 0,100 | 0,250 | 0,08333 | 0,000 | 0,453 | 0,175 | 0,375 | 0,000 |
| M 55-64 | 0,333 | 0,0000 | 0,100 | 0,250 | 0,08333 | 0,000 | 0,500 | 0,238 | 0,341 | 0,000 |
| M 65-74 | 0,200 | 0,6000 | 0,100 | 0,250 | 0,08333 | 0,000 | 0,421 | 0,389 | 0,342 | 0,250 |
| M 75-84 | 0,333 | 0,5000 | 0,100 | 0,167 | 0,05000 | 0,000 | 0,334 | 0,167 | 0,500 | 0,000 |
| M 85+ | 0,500 | 0,0000 | 0,100 | 0,125 | 0,05000 | 0,000 | 0,000 | 0,000 | 0,000 | 0,000 |
| F 25-34 | 0,000 | 0,0000 | 0,100 | 0,250 | 0,08333 | 0,000 | 0,000 | 0,000 | 0,000 | 0,000 |
| F 35-44 | 0,000 | 1,0000 | 0,100 | 0,250 | 0,08333 | 0,000 | 0,000 | 0,000 | 0,000 | 0,000 |
| F 45-54 | 0,000 | 0,0000 | 0,100 | 0,250 | 0,08333 | 0,000 | 0,500 | 0,334 | 0,500 | 0,000 |
| F 55-64 | 0,100 | 0,5000 | 0,100 | 0,250 | 0,08333 | 0,000 | 0,375 | 0,125 | 0,375 | 0,125 |
| F 65-74 | 0,091 | 0,4545 | 0,100 | 0,250 | 0,08333 | 0,000 | 0,375 | 0,334 | 0,500 | 0,167 |
| F 75-84 | 0,375 | 0,8750 | 0,100 | 0,167 | 0,05000 | 0,000 | 0,000 | 0,000 | 0,000 | 0,150 |
| F 85+ | 0,000 | 0,0000 | 0,100 | 0,125 | 0,05000 | 0,000 | 0,000 | 0,000 | 0,000 | 0,000 |

## Lipid lowering for primary prevention

- **Sources:**

Ariana survey 1997[8]

Ariana survey 2009[9]

- We assumed that Ariana surveys 1997 and 2009 are representative samples.

| **Statins for primary prevention (DG8)** | **1997** | **2009** |
| --- | --- | --- |
| M 25-34 | 0,000 | 0,01 |
| M 35-44 | 0,000 | 0,01 |
| M 45-54 | 0,000 | 0,07 |
| M 55-64 | 0,000 | 0,16 |
| M 65-74 | 0,000 | 0,11 |
| M 75-84 | 0,000 | 0,11 |
| M 85+ | 0,000 | 0,05 |
| F 25-34 | 0,000 | 0,02 |
| F 35-44 | 0,000 | 0,02 |
| F 45-54 | 0,000 | 0,05 |
| F 55-64 | 0,000 | 0,12 |
| F 65-74 | 0,000 | 0,16 |
| F 75-84 | 0,000 | 0,11 |
| F 85+ | 0,000 | 0,06 |

## Antihypertensive medication

- **Sources:**

Ariana survey 1997[8]

Ariana survey 2009[9]

- We assumed that Ariana surveys 1997 and 2009 are representative samples.

| **Antihypertensive medication (ADG9)** | **1997** | **2009** |
| --- | --- | --- |
| M 25-34 | 0,033 | 0,012 |
| M 35-44 | 0,033 | 0,014 |
| M 45-54 | 0,107 | 0,100 |
| M 55-64 | 0,295 | 0,288 |
| M 65-74 | 0,295 | 0,425 |
| M 75-84 | 0,295 | 0,381 |
| M 85+ | 0,295 | 0,281 |
| F 25-34 | 0,191 | 0,005 |
| F 35-44 | 0,091 | 0,054 |
| F 45-54 | 0,226 | 0,220 |
| F 55-64 | 0,334 | 0,387 |
| F 65-74 | 0,334 | 0,500 |
| F 75-84 | 0,334 | 0,431 |
| F 85+ | 0,234 | 0,300 |

# Sources

1. : **National Institute of Statistic:**

The National Institute of statistics represents a central organ in the national statistics system. According to its status, NIS is managed by a governing board chaired by the NIS Chief executive officer.

- Conduct regular surveys and census (every 10 years)
- Publish specific reports on demographic indicators:

Annual report and population projection up to 2024

(Online publication: [www.ins.nat.tn](http://www.ins.nat.tn) )

1. **: National Public Health Institute**
2. **: Tunisian Epidemiological Study on Acute Coronary Syndrome “TEPS-ACS 2009/2010**”: Survey in cardiology services for hospitals in the district of Tunis (M. Charles Nicolle. H. Rabta. H. Habib Thameur. H. Military and H. Abderrahman Mami L'Ariana) starting on November 2009. The study included all individuals without limitations of age or sex in hospital cardiology services of hospitals in the district of Tunis for a myocardial infarction in acute phase (first 28 days) in which the diagnosis was made according to the definition of myocardial infarction developed by WHO. The total number patients during the period of the study are 1178 patients.
3. : **National Medical Insurance (CNAM)**
4. : **National survey 2002-2003**: Survey in cardiology services for hospitals, the study included all individuals without limitations of age or sex admitted in hospital cardiology services. The total number patients are 48665 patients.
5. : **Regional survey 1997**: Survey in cardiology services for regional hospitals, the study included all individuals without limitations of age or sex admitted in hospital cardiology services.
6. ***:* Tunisian National Survey 2005(TAHINA):** The survey was cross-sectional from April to September 2005 and the target population was all 35-70 year old of both genders. With a total of 8007 subjects. It was based on a nationally representative stratified two-stage cluster sample of households according to the seven administrative regions of Tunisia.
7. ***:* Ariana survey 1997*:*** This study was conducted in 1997 on a sample of 5771 adults 35-65 years old in Ariana.
8. ***:*** **Ariana survey 2009**: This study was conducted in 2009 on a sample of 1340 adults 18-65 years old in Ariana.
9. : **Tunisian National survey, INSP 1996-1997:** This study was conducted in 1996 of a representative national sample of 5696 subjects aged 25 and over. Data were collected by means of a questionnaire in Arabic.
10. **: Tunisian National Nutrition Survey 1996/97:** The survey was cross-sectional from June 1996 to December 1997 on a nationally representative sample: 1735 households with a total of 3635 adults over 20 years old.
11. : **Survey AMI 2002:** In cardiology services for hospitals in the district of Tunis (M. Charles Nicolle. H. Rabta. H. Habib Thameur. H. Military. H. and H. Marsa Abderrahman Mami L'Ariana) during the period from 1 November 2001 to October 31. 2002. The study included all individuals without limitations of age or sex in hospital cardiology services of hospitals in the district of Tunis for a myocardial infarction in acute phase (first 28 days) in which the diagnosis was made according to the definition of myocardial infarction developed by WHO. The total number patients during the period of the study are 632 patients.
12. **PREMISE I 2002**: the survey of secondary prevention of myocardial infarction. Conducted in all patients followed in the outpatient cardiology. Internal medicine and neurology of the university hospitals in the district of Tunis. The study consists of patients who consulted during the period between October 2002 and February 2003 and fulfilling the inclusion criteria.

Included in the survey. patients (both sexes): the diagnosis is confirmed cardiovascular disease and / or cerebro-vascular defined by one or more of the following conditions: myocardial infarction. stable or unstable angina. stroke. transient ischemic attack; who received angioplasty. Carotid endarterectomy or cerebrovascular surgery.

- Having an age >= 21 years
- Taking outpatient treatment.
- With a duration of the disease more than one month and less than three years.

1. **PREMISE II 2010:** The survey of secondary prevention was conducted during the period between February 2010 and June 2010 in 600 patients followed in the outpatient cardiology of two university hospitals in the district of Tunis. Inclusion criteria:

- patients (both sexes) with confirmed diagnosis of cardiovascular disease and / or cerebrovascular disease defined by one or more of the following conditions: myocardial infarction, angina, stroke, CABG or PTCA, carotid endarterectomy or cerebrovascular surgery, hypertension, diabetes, dyslipidemia
- >= 21 years old
- receiving treatment uptake
- With a duration of the disease more than one month and less than three years.

# Appendix S2

# Clinical efficacy of interventions: relative risk reductions obtained from meta-analyses, and randomised clinical trials

| **Treatments** | **Relative risk reduction^†^** | **Comments** | **Source paper: First author (year), notes** |
| --- | --- | --- | --- |
| ***ST elevation myocardial infarction (STEMI)*** | | | |
|  |  |  |  |
| **Thrombolysis** | 31% (95% CI: 14,45) | <55 years: Odds Ratio (OR)=0.692; Relative Risk Reduction (RRR)=30.8% (95% CI: 14,45)  55-64 years: OR=0.736; RRR=26.4% (95% CI: 17,40)  65-74 years: OR=0.752; RRR=24.8% (95% CI: 15,37)  > 75 years: OR=0.844; RRR=15.6% (95% CI: 4,30) | Estess (2002)^1^ |
| **Aspirin** | 23% (95% CI: 15,30) | RRR=23% (95% CI: 15,30): outcome is vascular deaths | ISIS-2 (1988)^2^ |
| **Primary CABG surgery** | 39% (95% CI: 23,52) | OR=0.61 (95% CI: 0.48,0.77); RRR=39% (95% CI: 23,52) on page 565, 0-5 year mortality | Yusuf (1994)^3^ |
| **Primary PCI** | 30% (95% CI: 15,42) | OR=0.70 (95% CI: 0.58,0.85); RRR=30% (95% CI: 15,42) outcome compares primary angioplasty to thrombolytics. | Keeley (2003)^4^ |
| **Beta blockers** | 4% (95% CI: -8,15) | OR=0.96 (95% CI: 0.85,1.08); RRR=4% (95% CI: -8,15) on page 1732 | Freemantle (1999)^5^ |
| **ACE inhibitors** | 7% (95% CI: 2,11) | OR=0.93 (95% CI: 0.89,0.98); RRR=7% (95% CI: 2,11) for 30 day mortality in myocardial infarction | ACE Inhibitor Myocardial Infarction Collaborative Group (1998)^6^ |
| **Clopidogrel** | 3% (95% CI: 1,6) | RRR=3% (95% CI: 1,6) for 30 day mortality in myocardial infarction | Chen (2005)^7^  Sabatine (2005)^8^ |
| **Hospital CPR** | 33% (95% CI: 10,36) | Survival at 24 hours estimated to be 32%, discharge to home at 21%, and 1 year survival to be 15% overall. | Tunstall-Pedoe (1992)^9^  Nadkarni ^10^ |
| ***Non-ST-segment elevation acute coronary syndrome (NSTEACS):*** | | | |
|  |  |  |  |
| **Aspirin alone** | 15% (95% CI: 11,19) | OR=0.85 (95% CI: 0.49,0.95); RRR=15% (95% CI: 11,19). Outcome is vascular and nonvascular deaths on page 75. Assume appropriate for patients with NSTE-ACS. | Antithrombotic Trialists’ Collaboration (2002)^11^ |
| **Aspirin & heparin** | 33% (95% CI: -2,56) | OR=0.67 (95% CI: 0.48,1.02); RRR=33% (95% CI: -2,56%) in Table 2. The study outcome is composite MI death and non-fatal MI; compares those on aspirin & heparin to aspirin only. | Oler (1996)^12^ |
| **Platelet glycoprotein IIB/IIIA inhibitors** | 9% (95% CI: 2,16) | OR=0.91 (95% CI: 0.84,0.98); RRR=9% (95% CI: 2,16). Study looked at acute coronary syndrome without persistent ST elevation. | Boersma (2002)^13^ |
| **Early PCI** | 32% (95% CI: 5,51) | OR=0.68 (95% CI: 0.49,0.95); RRR=32% (95% CI: 5,51) | RITA 3 (Fox 2005)^14^ |
| **Primary CABG surgery** | 39% (95% CI: 23,52) | OR=0.61 (95% CI: 0.48,0.77); RRR=39% (95% CI: 23,52) on page 565, 0-5 year mortality | Yusuf (1994)^3^.  Assumed similar as STEMI. |
| **Clopidogrel** | 7% (95% CI: 2,11) | RRR=7% (95% CI: 2,11) | Yusuf (2001)^15^ |
| **Beta blockers** | 4% (95% CI: -8,15) | OR=0.96 (95% CI: 0.85,1.08); RRR=4% (95% CI: -8,15) on page 1732 | Freemantle (1999)^5^  Assumed similar as STEMI. |
| **ACE inhibitors** | 7% (95% CI: 2,11) | OR=0.93 (95% CI: 0.89,0.98); RRR=7% (95% CI: 2,11) for 30 day mortality in myocardial infarction | ACE Inhibitor Myocardial Infarction Collaborative Group (1998)^6^ |
| ***Secondary prevention post myocardial infarction/revascularisation:*** | | | |
|  |  |  |  |
| **Aspirin** | 15% (95% CI: 11,19) | OR=0.85 (95% CI: 0.49,0.95); RRR=15% (95% CI: 11,19). Outcome is vascular and nonvascular deaths on page 75. This data seems to be appropriate to this outcome in CHD patients. | Antithrombotic Trialists’ Collaboration (2002)^11^ |
| **Beta blockers** | 23% (95% CI: 15,31) | OR=0.77 (95% CI: 0.69,0.85); RRR=23% (95% CI: 15,31) on page 1734. Odds of death in long term trials. | Freemantle (1999)^5^ |
| **ACE inhibitors or Angiotensin-II receptor antagonists** | 20% (95% CI: 13,26) | OR=0.80 (95% CI: 0.74,0.87); RRR=20% (95% CI: 13,26) on page 1577, death up to four years [endpoint of study looking at those with heart failure or LV dysfunction]. | Flather (2000)^16^ |
| **Statins** | 24% (95% CI: 10,26) | RRR=24% (95% CI: 10,26)  Intensive statin therapy in acute coronary syndromes. | Hulten (2006)^17^ |
| **Warfarin** | 22% (95% CI: 13,31) | OR=0.78 (95% CI: 0.67,0.90); RRR=22% (95% CI: 10,33) | Anand and Yusuf (1999)^18^ |
| **Rehabilitation** | 26% (95% CI: 10,39) | OR=0.74 (95% CI: 0.61,0.90); RRR=26% (95% CI: 10,39) in Figure 1, page 685 Taylor reference | Taylor (2004)^19^ |
| ***Chronic stable coronary artery disease:*** | | | |
|  |  |  |  |
| **CABG surgery**  **years 0-5** | 39% (95% CI:23,52) | OR = 0.61 (95% CI: 0.48-0.77), RRR 39% (95% CI: 23,52) on page 565, 5 year mortality | Yusuf (1994)^3^ |
| **CABG surgery**  **years 6-10** | 32% (95% CI: 2,30) | OR = 0.83 (95% CI: 0.70-0.98), RRR 17% (95% CI: 2,30) on page 565, 10 year mortality.  OR = 0.68 (95% CI: 0.56-0.83), RRR 32% (95% CI: 17,44) on page 565, 7 year mortality  CABG compared to medical treatment | Yusuf (1994)^3^ |
| **Angioplasty** | No effect |  | Boden (2007) ^20^ |
| **Aspirin** | 15% (95% CI: 11,19) | OR=0.85 (95% CI: 0.49-0.95); RRR=15% (95% CI: 11,19). Outcome is vascular and nonvascular deaths on page 75. | Antithrombotic Trialists’ Collaboration (2002)^35^ |
| **Statins** | 23% (95% CI: 10,26) | RRR=23% (95% CI 10,26)  Standard dose statin therapy in coronary artery disease. | Wilt (2004)^21^ |
| **ACE inhibitors/ARB** | 17% (95% CI: 6,28) | RRR=17% (95% CI 6,28) | Al-Mallah (2006)^22^ |
| ***Heart failure in patients requiring hospitalisation or in the community:*** | | | |
|  |  |  |  |
| **ACE inhibitors** | 20% (95% CI: 13,26) | OR=0.80 (95% CI: 0.74,0.87); RRR=20% (95% CI: 13,26) on page 1577 [death up to four years was study endpoint for those with heart failure or LV dysfunction] | Flather (2000)^16^ |
| **Beta blockers** | 35% (95% CI: 26,43) | OR=0.65 (95% CI: 0.57,0.74); RRR=35% (95% CI: 26,43): all cause mortality | Shibata (2001)^23^ |
| **Spironolactone** | 30% (95% CI: 18,41)  31% (95% CI: 18,42) | OR=0.70 (95% CI: 0.59,0.82); RRR=30% (95% CI: 18,41) in those that had at least one cardiac related hospitalisation.  OR=0.69 (95% CI: 0.58,0.82); RRR=31% (95% CI: 18,42) in entire study population consisting of those with community heart failure, page 711. | Pitt (1999)^24^ |
| **Aspirin** | 15% (95% CI: 11,19) | OR=0.85 (95% CI: 0.49,0.95); RRR=15% (95% CI: 11,19). Outcome is vascular and nonvascular deaths on page 75. | Antithrombotic Trialists’ Collaboration (2002)^11^ |
| **Statins** | No effect |  | Kjekshus (2007)^25^  Tavazzi (2008)^26^ |
| ***Primary prevention therapies:*** | | | |
|  |  |  |  |
| **Treatments for high blood pressure** | 13% (95% CI: 6,19) | OR=0.87 (95% CI: 0.81,0.94); RRR=13% (95% CI: 6,19) in those with high blood pressure without disease at entry. [RRR=29% (95% CI: 17,37) those with average blood pressure and CHD, treated with ACE inhibitors] | Law (2003)^27^ |
| **Statins** | 35% (95% CI: 11,52) | OR=0.65 (95% CI: 0.48,0.89); RRR=35% (95% CI: 11,52) for CHD mortality (only trials using statins), Figure 3 on page 4 | Pignone (2000)^28^ |

^†^Relative risk reduction (RRR) calculated as 1 – odds ratio

# Beta coefficients for major risk factors

**Estimated β coefficients from multiple regression analyses for the relationship between absolute changes in population mean risk factors and percentage changes in coronary heart disease mortality for men and women, stratified by age. Data sources, values and comments.**

| **Systolic blood pressure** | | **Age group (years)** | | | | |
| --- | --- | --- | --- | --- | --- | --- |
|  | | **25-44** | **45-54** | **55-64** | **65-74** | **75+** |
|  | |  |  |  |  |  |
| **Men** (hazard ratio per 20 mmHg) | | 0.49 | 0.49 | 0.52 | 0.58 | 0.65 |
| Men (log hazard ratio per 1 mmHg) | | **-0.036** | **-0.035** | **-0.032** | **-0.027** | **-0.021** |
|  | |  |  |  |  |  |
| *Minimum* | | *-0.029* | *-0.028* | *-0.026* | *-0.022* | *-0.017* |
| *Maximum* | | *-0.043* | *-0.042* | *-0.039* | *-0.032* | *-0.025* |
|  | |  |  |  |  |  |
|  | |  |  |  |  |  |
| **Women** (hazard ratio per 20 mmHg) | | 0.40 | 0.40 | 0.49 | 0.52 | 0.59 |
| Women (log hazard ratio per 1 mmHg) | | **-0.046** | **-0.046** | **-0.035** | **-0.032** | **-0.026** |
| *Minimum* | | *-0.037* | *-0.037* | *-0.028* | *-0.026* | *-0.021* |
| *Maximum* | | *-0.055* | *-0.055* | *-0.042* | *-0.039* | *-0.031* |
|  | |  |  |  |  |  |
|  | |  |  |  |  |  |
| Source: Prospective studies collaborative meta-analysis, Lancet 2002^53^ | | | | | | |
| Units: Percentage change in CHD mortality per 20 mmHg change in systolic blood pressure | | | | | | |
| **Strengths:** | Large dataset, includes US data, adjusted for regression dilution bias, consistent with randomised controlled trials, results stratified by age and sex, with 95% confidence intervals | | | | | |
| **Limitations:** | Some publication bias still possible | | | | | |

| **Cholesterol** | **Age groups (years)** | | | | | | |
| --- | --- | --- | --- | --- | --- | --- | --- |
|  | **25-44** | | **45-54** | **55-64** | **65-74** | **75-84** | **85+** |
| **Mortality reduction per 1 mmol/l** | | | | | | | |
| Men | 0.55 | | 0.53 | 0.36 | 0.21 | 0.21 | 0.21 |
| Women | 0.57 | | 0.52 | 0.35 | 0.23 | 0.23 | 0.23 |
| **Log coefficient** | | | | | | | |
| **Men** | **-0.799** | | **-0.755** | **-0.446** | **-0.236** | **-0.117** | **-0.083** |
| *Minimum* | *-0.639* | | *-0.604* | *-0.357* | *-0.189* | *-0.093* | *-0.067* |
| *Maximum* | *-0.958* | | *-0.906* | *-0.536* | *-0.283* | *-0.140* | *-0.100* |
|  |  | |  |  |  |  |  |
| **Women** | **-0.844** | | **-0.734** | **-0.431** | **-0.261** | **-0.174** | **-0.051** |
| *Minimum* | *-0.675* | | *-0.587* | *-0.345* | *-0.209* | *-0.139* | *-0.041* |
| *Maximum* | *-1.013* | | *-0.881* | *-0.517* | *-0.314* | *-0.209* | *-0.062* |
| Source: Prospective studies collaborative meta-analysis, Lancet 2007^29^ | | | | | | | |
| Units: | | Percentage change in CHD mortality per 1 mmol/l change in total cholesterol | | | | | |
| **Strengths:** | | Includes US data, adjusted for regression dilution bias, includes randomised controlled trials, RCT values consistent with observational data, results stratified by age and sex, with 95% confidence intervals | | | | | |
| **Limitations:** | | Some publication bias still possible | | | | | |

| **Body Mass Index (BMI)** | | **Age groups (years)** | | | | |
| --- | --- | --- | --- | --- | --- | --- |
|  | | **<44** | **45-59** | **60-69** | **70-79** | **80+** |
| *James et.al (2004):* | |  |  |  |  |  |
| Hazard ratio | | 0.89 | 0.91 | 0.95 | 0.96 | 0.97 |
| Risk reduction† per 1 kg/m^2^ | | 0.11 | 0.09 | 0.05 | 0.04 | 0.03 |
| Age gradient (45-59 as reference) | | 1.22 | **1.00** | 0.56 | 0.44 | 0.33 |
| *Bogers (2006):*  Relative risks, CHD deaths per 5 BMI units (kg/m^2^) | |  | **1.16** |  |  |  |
| Relative risks per 1 kg/m^2^ applying age gradients from James et.al | | 1.04 | 1.03 | 1.02 | 1.01 | 1.01 |
| **Log coefficients** | | **0.0363** | **0.0297** | **0.0165** | **0.0132** | **0.0099** |
| *Minimum* | | *0.0255* | *0.0209* | *0.0116* | *0.0093* | *0.0070* |
| *Maximum* | | *0.0466* | *0.0381* | *0.0212* | *0.0169* | *0.0127* |
| Source: Bogers et al (2006)^55^, James et al (2004)^56^ | | | | | | |
| Units: | Percentage change in CHD mortality per 1 kg/m^2^ change in BMI | | | | | |
| **Strengths:** | Large number of studies included. Adjusted for blood pressure, total cholesterol, and physical activity. 95% confidence intervals included. | | | | | |
| **Limitations:** | Observational data; age gradient applied from James study | | | | | |

^†^ Risk reduction = 1 – hazard ratio

**REFERENCES:**

(1) Estess JM, Topol EJ. Fibrinolytic treatment for elderly patients with acute myocardial infarction. Heart 2002 April;87:308-11.

(2) ISIS-2 (Second international study of infarct survival) collaborative group. Randomised trial of intravenous streptokinase, oral aspirin, both, or neither among 17 187 cases of suspected acute myocardial infarction: ISIS-2. Lancet 1988;8607:349-60.

(3) Yusuf S, Zucker D, Peduzzi P, Fisher LD, Takaro T, Kennedy JW et al. Effect of Coronary-Artery Bypass Graft-Surgery on Survival - Overview of 10-Year Results from Randomized Trials by the Coronary-Artery Bypass Graft-Surgery Trialists Collaboration. Lancet 1994;344:563-70.

(4) Keeley EC, Boura JA, Grines CL. Primary angioplasty versus intravenous thrombolytic therapy for acute myocardial infarction: a quantitative review of 23 randomised trials. Lancet 2003;361:13-20.

(5) Freemantle N, Cleland J, Young P, Mason J, Harrison J. beta Blockade after myocardial infarction: systematic review and meta regression analysis. BMJ 1999 June 26;318:1730-7.

(6) Ace Inhibitor Myocardial Infarction Collaborative Group. Indications for ACE inhibitors in the early treatment of acute myocardial infarction: Systematic overview of individual data from 100 000 patients in randomized trials. Circulation 1998;97:2202-12.

(7) Chen ZM, Jiang LX, Chen YP, et al. Addition of clopidogrel to aspirin in 45,852 patients with acute myocardial infarction: randomised placebo-controlled trial. Lancet 2005;366:1607-21.

(8) Sabatine MS, Cannon CP, Gibson CM, Lopez-Sendon JL, Montalescot G, Theroux P et al. Addition of clopidogrel to aspirin and fibrinolytic therapy for myocardial infarction with ST-segment elevation. New England Journal of Medicine 2005;352:1179-89.

(9) Tunstall-Pedoe H, Bailey L, Chamberlain DA, Marsden AK, Ward ME, Zideman DA. Survey of 3765 cardiopulmonary resuscitations in British hospitals (the BRESUS Study): methods and overall results. BMJ 1992 May 23;304:1347-51.

(10) Nadkarni VM, Larkin GL, Peberdy MA, Carey SM, Kaye W, Mancini ME et al. First documented rhythm and clinical outcome from in-hospital cardiac arrest among children and adults. JAMA 2006 January 4;295:50-7.

(11) Antithrombotic Trialists' Collaboration. Collaborative meta-analysis of randomised trials of antiplatelet therapy for prevention of death, myocardial infarction, and stroke in high risk patients (vol 324, pg 71, 2002). British Medical Journal 2002;324:141.

(12) Oler A, Whooley MA, Oler J, Grady D. Adding heparin to aspirin reduces the incidence of myocardial infarction and death in patients with unstable angina. A meta-analysis. JAMA 1996 September 11;276:811-5.

(13) Boersma E, Harrington RA, Moliterno DJ, White H, Theroux P, Van de Werf F et al. Platelet glycoprotein IIb/IIIa inhibitors in acute coronary syndromes: a meta-analysis of all major randomised clinical trials. Lancet 2002;359:189-98.

(14) Fox KAA, Poole-Wilson P, Clayton TC, Henderson RA, Shaw TRD, Wheatley DJ et al. 5-year outcome of an interventional strategy in non-ST-elevation acute coronary syndrome: the British Heart Foundation RITA 3 randomised trial. Lancet 2005;366:914-20.

(15) Yusuf S, Zhao F, Mehta SR, Chrolavicius S, Tognoni G, Fox KK. Effects of clopidogrel in addition to aspirin in patients with acute coronary syndromes without ST-segment elevation. New England Journal of Medicine 2001;345:494-502.

(16) Flather MD, Yusuf S, Kober L, Pfeffer M, Hall A, Murray G et al. Long-term ACE-inhibitor therapy in patients with heart failure or left-ventricular dysfunction: a systematic overview of data from individual patients. ACE-Inhibitor Myocardial Infarction Collaborative Group. Lancet 2000 May 6;355:1575-81.

(17) Hulten E, Jackson JL, Douglas K, George S, Villines TC. The effect of early, intensive statin therapy on acute coronary syndrome: a meta-analysis of randomized controlled trials. Arch Intern Med 2006;166:1814-21.

(18) Anand SS, Yusuf S. Oral anticoagulant therapy in patients with coronary artery disease: a meta-analysis. JAMA 1999 December 1;282:2058-67.

(19) Taylor RS, Brown A, Ebrahim S, Jolliffe J, Noorani H, Rees K et al. Exercise-based rehabilitation for patients with coronary heart disease: systematic review and meta-analysis of randomized controlled trials. Am J Med 2004 May 15;116:682-92.

(20) Boden WE, O'Rourke RA, Teo KK, Hartigan PM, Maron DJ, Kostuk WJ et al. Optimal medical therapy with or without PCI for stable coronary disease. New England Journal of Medicine 2007;356:1503-16.

(21) Wilt TJ, Bloomfield HE, MacDonald R, et al. Effectiveness of statin therapy in adults with coronary heart disease. Arch Intern Med 2004;164:1427-36.

(22) Al-Mallah MH, Tleyjeh IM, Abdel-Latif AA, Weaver WD. Angiotensin-converting enzyme inhibitors in coronary artery disease and preserved left ventricular systolic function: a systematic review and meta-analysis of randomized controlled trials. Journal of the American College of Cardiology 2006;47:1576-83.

(23) Shibata MC, Flather MD, Wang DL. Systematic review of the impact of beta blockers on mortality and hospital admissions in heart failure. European Journal of Heart Failure 2001;3:351-7.

(24) Pitt B, Zannad F, Remme WJ, Cody R, Castaigne A, Perez A et al. The effect of spironolactone on morbidity and mortality in patients with severe heart failure. New England Journal of Medicine 1999;341:709-17.

(25) Kjekshus J, Apetrei E, Barrios V, et al. Rosuvastatin in older patients with systolic heart failure. New England Journal of Medicine 2007;357:2248-61.

(26) Tavazzi L, Maggioni AP, Marchioli R, et al. Effect of rosuvastatin in older patients with chronic heart failure (the GISSI-HF trial): a randomised, double-blind, placebo-controlled trial. Lancet 2008;372:1231-9.

(27) Law M, Wald N, Morris J. Lowering blood pressure to prevent myocardial infarction and stroke: a new preventive strategy. Health Technol Assess 2003;7:1-94.

(28) Pignone M, Phillips C, Mulrow C. Use of lipid lowering drugs for primary prevention of coronary heart disease: meta-analysis of randomised trials. British Medical Journal 2000;321:983-6.

(29) Lewington S, Clarke R, Qizilbash N, Peto R, Collins R. Age-specific relevance of usual blood pressure to vascular mortality: a meta-analysis of individual data for one million adults in 61 prospective studies. Lancet 2002 December 14;360:1903-13.

(30) Lewington S, Whitlock G, Clarke R, Sherliker P, Emberson J, Halsey J et al. Blood cholesterol and vascular mortality by age, sex, and blood pressure: a meta-analysis of individual data from 61 prospective studies with 55000 vascular deaths. Lancet 2007;370:1829-39.

(31) Bogers RP, Hoogenveen RT, Boshuizen H, Woodward M, Knekt P, Van Dam RM et al. Overweight and obesity increase the risk of coronary heart disease: A pooled analysis of 30 prospective studies. European Journal of Epidemiology 2006;21:313.

(32) James WPT, Jackson-Leach R, Mhurchu CN et al. Overweight and obesity (high body mass index). In: Ezatti M, Lopez AD, Rodgers A, Murray CJL, editors. Comparative quantification of risk. Global and regional burden of disease attributable to selected major risk factors. Volume 1 ed. World Health Organization; 2004. p. 497-596.
